# Cholesterol sulfate replenishment rejuvenates aged hematopoietic stem cell phenomes

**DOI:** 10.7150/thno.126134

**Published:** 2026-03-17

**Authors:** Yunyu Feng, Ning Li, Nan Wang, Wei He, Hongjian Li, Xue Cui, Bochuan Wang, Runkuan Qin, Huandi Qiu, Qiang Qiu, Li Zheng, Yuanyuan Sun, Linye He, Cong Pan, Anping Su, Zhihui Li, Yiguo Hu

**Affiliations:** 1 State Key Laboratory of Biotherapy, West China Hospital, Sichuan University, Chengdu, China.; 2 Department of Clinical Translational Innovation Center and Molecular Medicine Research Center, West China Hospital, Sichuan University, Chengdu, China.; 3 Institute for Immunology and School of Medicine, Tsinghua University, Beijing, China.; 4 Department of Hematology and Research Laboratory of Hematology, West China Hospital, Sichuan University, Chengdu, China.; 5 Institute of Cardiovascular Surgery, West China Hospital, Sichuan University, Chengdu, China.; 6 Laboratory of thyroid and parathyroid disease, Frontiers Science Center for Disease-related Molecular Network, West China Hospital, Sichuan University, Chengdu, China.; 7 Department of Thyroid Surgery, West China Hospital, Sichuan University, Chengdu, China.; 8 School of Biological Sciences, Guizhou Education University, Guiyang, China.; 9 Translational Medicine Research Center, eBond Pharmaceutical Technology Co., Ltd., Chengdu, China.

**Keywords:** Aging, hematopoietic stem cells (HSCs), Cholesterol sulfate (CS), rejuvenation, DNA damage

## Abstract

The aging of hematopoietic stem cells (HSCs) impairs their regenerative and self-renewal capacities, contributing to immune dysfunction and hematologic diseases, yet safe and effective rejuvenation strategies remain elusive.

**Methods:**

This study employed a human data-driven, metabolism-centered approach to identify endogenous compounds with rejuvenating potential for aged HSCs. Genome-scale metabolic models and single-cell transcriptomic analyses were used to characterize metabolic changes in human and mouse HSCs. Aged mice were treated with cholesterol sulfate (CS), and HSC functionality was assessed via proliferation assays, DNA damage analysis, and bone marrow transplantation. Molecular docking and *in vivo* experiments further explored the role of the nuclear receptor RORA.

**Results:**

Supplementation with CS in aged mice enhanced HSC proliferation, reduced DNA damage, and restored hematopoietic output. Metabolic modeling revealed increased CS hydrolysis as a key alteration in aged HSCs, while single-cell transcriptomics showed that CS treatment suppressed inflammatory signaling and eliminated stress-responsive HSC subpopulations. Mechanistically, CS was identified as a potent ligand that activates RORA. Functional studies suggested that RORA functions as a key mediator in this process.

**Conclusion:**

CS supplementation reverses aging-associated HSC decline by enhancing proliferation, reducing DNA damage, and modulating inflammatory pathways, with RORA serving as an essential mediator. These findings highlight the CS-RORA axis as a promising therapeutic target for rejuvenating aged HSCs and preserving hematopoietic function.

## Introduction

Aging is the progressive, systemic deterioration of biological function and stress resilience, culminating in organ dysfunction and elevated mortality risk 1. The hematopoietic system is particularly vulnerable to aging, as it relies on hematopoietic stem cells (HSCs) to sustain lifelong blood and immune cell production. Age-associated HSC dysfunction manifests as impaired immune competence, anemia, myeloid lineage bias, and an increased incidence of hematological malignancies, including chronic myeloid leukemia (CML) and myelodysplastic syndromes (MDS) 2, 3. Hematopoietic aging is driven by multiple interconnected processes, including genomic instability, telomere attrition, and mitochondrial dysfunction, collectively leading to the functional exhaustion of HSCs 4, 5. Clinically, this decline is exemplified by the reduced efficacy of bone marrow transplantation (BMT) from elderly donors, underscoring the urgent need to identify strategies that restore HSC function during aging 6-9.

While hematopoietic aging is evolutionarily conserved, most mechanistic insights have been derived from murine models 3, 10, 11. Fundamental differences between mouse and human HSCs, particularly in telomere dynamics, transcriptional regulation, and stress responses, limit the direct translational relevance of these findings 12. Recent advances in high-throughput sequencing and systems-level analyses have enabled comprehensive interrogation of human hematopoiesis at unprecedented resolution 2, 13, 14. However, converting descriptive human aging signatures into actionable interventions that restore HSC function with clear mechanisms and acceptable safety remains a major challenge.

Metabolic regulation has emerged as a central determinant of stem cell aging and tissue regeneration 15. Consistent with a causal role for metabolism in hematopoietic aging, a recent metabolic atlas identified uridine as a metabolite capable of rejuvenating aged HSCs *in vivo* 16. However, many pharmacological interventions targeting aging rely on repurposed drugs with systemic toxicity or poorly defined mechanisms, such as rapamycin, metformin, and senolytics 17-20. In contrast, endogenous metabolites may provide a more physiological and potentially better-tolerated modality for modulating aging-associated dysfunction. While several endogenous compounds, such as NAD⁺ precursors and α-ketoglutarate, have shown promise in delaying age-related decline 21-23, a systematic, human-data-guided framework to identify metabolites that rejuvenate aged human HSCs is still lacking.

To address this gap, we established a human data-driven, metabolism-centered strategy to systematically identify endogenous compounds with rejuvenating potential in aged HSCs. Through this pipeline, we identified Cholesterol Sulfate (CS), a sulfated sterol metabolite, as a key molecule specifically depleted in aged HSCs. While previously known for its roles in skin barrier function and membrane stability, our findings reveal a surprising and pivotal function of CS in maintaining HSC homeostasis. Supplementation with CS reversed aging-associated phenotypes, enhancing proliferation, reducing DNA damage, and eliminating stress-responsive cell populations. Mechanistically, we uncovered activation of the nuclear receptor RORA as a key downstream effector of CS signaling, linking sterol metabolism to the transcriptional control of HSC homeostasis. These findings establish the CS-RORA axis as a novel, targetable pathway for restoring hematopoietic health and define endogenous metabolic regulation as a promising therapeutic avenue for rejuvenation.

## Materials and Methods

### Ethics statement and experimental animals

Human sample collection and analysis were approved by the Ethics Committee on Biomedical Research, West China Hospital of Sichuan University (Approval No. 1020). Written informed consent was obtained from all donors. All animal procedures adhered strictly to the guidelines established by the Animal Care and Use Committee of the State Key Laboratory of Biotherapy, Sichuan University (Approval No. 2021967A). The C57BL/6J (CD45.2) strain was sourced from the Jackson Laboratory (Bar Harbor, ME). Animal husbandry was conducted within the specialized facility of the State Key Laboratory of Biotherapy under standard conditions. To minimize experimental variability, study groups comprised age- and sex-matched littermates, with young cohorts aged 8-12 weeks and aged cohorts at 12-24 months.

### Generation of conditional knockout models

To assess the functional necessity of RORA, a hematopoietic-specific knockout line was established by crossing *Rora^fl/fl^* mice (generated on a C57BL/6J background by Biocytogen, Beijing) with *Mx1-Cre* transgenic mice (Jackson Laboratory). Genetic deletion was induced in 8-week-old *Rora^fl/fl^ Mx1-Cre* progeny via intraperitoneal (IP) administration of polyinosinic:polycytidylic acid (pIpC) at 15 mg/kg. The induction regimen comprised five doses administered every other day. Littermate *Rora^fl/fl^* controls received an identical pIpC regimen. HSCs were isolated for phenotypic analysis at 4- and 12-weeks post-induction.

### Pharmacological interventions and irradiation models

For rejuvenation studies, aged mice (1-2 years old) received twice-daily IP injections of either CS (25 mg/kg, n = 3) or vehicle (DMSO, n = 3) for 14 days. To delineate the differential impact of CS versus supraphysiological mTOR activation, a mechanistic comparison was performed. C57BL/6 mice (n = 4-5 per group) were randomized into three cohorts and subjected to a short-term (3-day) regimen: CS treatment (25 mg/kg); treatment with the mTOR agonist MHY1485 (5 mg/kg); and vehicle control. Following the 3-day window, LSK (Lin^low^-Sca1^+^-c-Kit^+^) cells were purified by FACS to evaluate mTOR pathway activation markers via immunofluorescence.

To evaluate DNA repair dynamics, a sublethal irradiation model was employed. Young mice were pre-conditioned with three doses of PBS or CS (25 mg/kg, IP, 12 h intervals) prior to exposure to 2 Gy γ irradiation. Bone marrow was harvested at 1, 2, or 6 h post-irradiation for subsequent γH2AX foci quantification and comet assays.

### Flow cytometry and cell sorting

Hematopoietic populations were analyzed and isolated with BD FACSAria III or BD Fortessa X instruments. Data were processed with FlowJo software (v10). Bone marrow cells were flushed from femurs and tibias, followed by red blood cell lysis. For HSC enrichment, lineage-positive cells were depleted. This was achieved using the Lineage Cell Depletion Kit (Miltenyi Biotec) with anti-PE microbeads designed to target a cocktail of lineage markers (Mac-1, Gr-1, Ter-119, B220, Il-7Rα, CD3, CD4, CD8). The resulting lineage-negative fraction was stained with fluorophore-conjugated antibodies to define specific progenitor subsets. Detailed antibody panels and surface marker definitions are provided in Table S1 24-26. Propidium iodide was utilized to exclude non-viable cells during sorting.

### Single-cell gel electrophoresis

DNA strand breaks were quantified with the Trevigen Comet Assay kit. Briefly, sorted HSCs were suspended in low-melting-point agarose and layered onto CometSlides to ensure uniform processing. Cells were lysed overnight at 4 °C, followed by alkaline unwinding to reveal single-strand breaks. Electrophoresis was conducted under alkaline conditions. DNA tails were visualized by SYBR Green staining. Comet metrics, including Tail DNA% and Olive Tail Moment, were calculated to assess genomic integrity.

### Cell culture

HEK293T cells were maintained in DMEM supplemented with 10% fetal bovine serum (FBS) and 1% penicillin/streptomycin in a humidified 37 °C incubator.

### Immunofluorescence microscopy

Purified HSCs were subjected to a 30-min fixation using BD Cytofix Buffer at ambient temperature. Following fixation, cells were adhered to fibronectin-precoated glass slides. After PBS washing, cells were permeabilized with 0.2% Triton X-100 and blocked with 10% Donkey Serum (Sigma-Aldrich) to minimize non-specific binding. Primary antibodies targeting key proteins (detailed in Table S2) were incubated for 1 h, followed by fluorophore-conjugated secondary antibodies. Nuclei were counterstained with DAPI included in the ProLong Gold Antifade Mountant (Thermo Fisher). Confocal microscopy was executed utilizing Zeiss LSM710 and AxioObserver Z1 systems. High-resolution captures were obtained with 633 nm objectives. Image analysis was conducted using AxioVision 4.6 software. Image deconvolution and 3D reconstruction were accomplished with Volocity software (v6.0, Perkin Elmer), and quantitative analysis of Mean Fluorescence Intensity (MFI) was executed using ZEN software.

### Transcriptomic data mining and metabolic modeling

A comprehensive HSC transcriptomic dataset was curated by querying the Gene Expression Omnibus (GEO) repository (https://www.ncbi.nlm.nih.gov/geo/), with strict donor stratification by age. Metabolic phenotypes were simulated through the construction of context-specific Genome-Scale Metabolic Models (GEMs) utilizing the ftINIT algorithm in MATLAB R2023a 27, 28. For bulk RNA-seq data, transcript abundance was quantified and converted to Transcripts Per Million (TPM) to normalize for gene length and sequencing depth. Similarly, scRNA-seq data from HSCs were preprocessed and transformed into TPM values to ensure consistency across datasets. These TPM-converted transcriptomes were integrated into ftINIT, which leverages metabolic network constraints and expression profiles to generate cell-specific GEMs. Human- and mouse-GEM frameworks were downloaded to provide the reference metabolic reaction networks. Models were analyzed to infer metabolic differences linked to HSC states 28, 29. The heatmaps were generated with the pheatmap R package (v1.0.12) 30.

### Single-cell library preparation and sequencing

Following FACS sorting, single HSCs were partitioned into nanoliter-scale gel beads-in-emulsion with the 10x Genomics Chromium Controller. Libraries were constructed utilizing the Chromium Single Cell 3' Library and Gel Bead Kit v2, following the manufacturer's standard instructions, with a target recovery of approximately 10,000 cells per sample. cDNA synthesis and amplification (12 cycles) were performed on a Bio-Rad C1000 Touch thermal cycler. Library quality control, including fragment size distribution, was assessed on a Fragment Analyzer (Advanced Analytical) with the High Sensitivity NGS Analysis Kit. Final libraries were quantified via qPCR (Kapa Biosystems), normalized to 2 nM, and sequenced on an Illumina NovaSeq 6000 platform.

### Bulk RNA-seq pipeline

Total RNA was extracted from sorted populations with Trizol (Thermo Fisher Scientific), followed by poly(A) mRNA enrichment from 1 μg of total RNA input (NEBNext Magnetic Isolation Module). Stranded RNA libraries were constructed utilizing the NEBNext Ultra Directional RNA Library Prep Kit, followed by sequencing on the Illumina HiSeq X Ten system (150 bp paired-end). To remove adapter sequences and filter out low-quality bases (Phred score < 20), Trim Galore (v0.2.7) was employed for raw read processing. The alignment of high-quality reads to the mm10 reference genome was performed by Hisat2 (v0.2.4) 31, and gene counts were generated via HTSeq (v0.9.0) 32.

### Single-cell RNA-seq data analysis

Primary sequencing outputs were processed using Cell Ranger (v6.0.1) for demultiplexing and alignment against the mm10 reference genome. Downstream analysis, including quality filtering and cell clustering, was executed within the R environment (v4.1.0) utilizing the Seurat package (v4.0.5) 33. To ensure high-quality data, rigorous cell-filtering criteria were applied. Only those cells containing more than 500 UMIs and 250 detected genes were considered. Furthermore, any cells with a mitochondrial read proportion exceeding 20% were excluded from the final analysis. Data were log-normalized, and batch effects were corrected through Canonical Correlation Analysis (CCA) on the top 2,000 variable features. Dimensionality reduction was achieved via Principal Component Analysis (PCA), with selection of PCs that contributed more than 5% standard deviation or accounted for 90% of the cumulative variance. Clusters were defined via graph-based clustering and visualized within Uniform Manifold Approximation and Projection (UMAP) space. Cell identity was assigned based on canonical lineage markers 26.

### Trajectory inference and functional scoring

Differentiation dynamics were modeled with Monocle3 (v1.0.0) 34 to reconstruct HSC differentiation trajectories. Cells were ordered along a pseudotemporal trajectory, with LT-HSCs set as the root. Genes that varied in expression in correlation to pseudotime were found using the “*graph_test*” function in Monocle3. Genes were grouped by k-means clustering (k = 4) and visualized along pseudotime. To quantify pathway activation at the single-cell level, UCell scores (v1.1.0) were calculated for aging-related gene signatures curated from the MSigDB H, C2, and C5 collections 35.

### Differential analysis and enrichment analysis

Platform-specific statistical frameworks were utilized to perform differential expression analysis. Seurat *FindMarkers* (Wilcoxon rank-sum test) for scRNA-seq was applied (log2FC > 0.263, P < 0.05). RNA-seq and microarray data were processed through the DESeq2 (v1.32.0) 36 and limma (v3.48.3) 37 packages, respectively, where a cutoff of |log2FC| > 1 and P < 0.05 was maintained. The Benjamini-Hochberg procedure was used to control the false discovery rate (FDR). Functional enrichment was assessed via Fisher's exact test for Gene Ontology (GO) terms 38 and fgsea (v1.18.0) for Gene Set Enrichment Analysis (GSEA).

### Protein and ligand preparation and molecular docking

The three-dimensional structure of CS was downloaded from the PubChem database (CID: 636741). The crystal structure of the human RORα ligand-binding domain (LBD) was retrieved from the Protein Data Bank (PDB ID: 4S15), which contains a co-crystallized sterol-like ligand (4ACD8).

Protein and ligand structures were prepared in MGLTools (v1.5.7), including removal of water molecules, addition of polar hydrogens, calculation of Gasteiger charges, and definition of rotatable bonds. The docking site was defined as a 10 Å radius region centered on the co-crystallized ligand. Molecular docking was executed with AutoDock Vina (v1.1.2) under default parameters. Multiple independent docking runs were carried out, and the conformation with the lowest predicted binding free energy was selected for subsequent molecular dynamics simulations.

### Molecular dynamics simulation and analysis

Molecular dynamics (MD) simulations were conducted in GROMACS (v2024.03) 39 to evaluate the stability and interaction dynamics of the RORA-ligand complexes. The Amber14SB force field 40 was applied to the protein, whereas ligand parameters and topologies were generated with ACPYPE based on the General Amber Force Field (GAFF). The complex was solvated in a dodecahedral box with TIP3P water molecules 41, and neutralized by adding Na⁺ and Cl⁻ ions to achieve a physiological concentration of 0.150 mol/L.

Energy minimization was performed with the steepest descent algorithm for 50,000 steps to remove steric clashes. The system was equilibrated sequentially under 100 ps under constant number, volume, and temperature (NVT) conditions, followed by 100 ps under constant number, pressure, and temperature (NPT), during which positional restraints were applied to protein heavy atoms. Temperature and pressure were maintained at 300 K and 1 bar via the V-rescale thermostat and Parrinello-Rahman barostat, respectively. Production simulations were run for 100 ns with a 2-fs integration timestep and without restraints. Long-range electrostatics were treated with the Particle Mesh Ewald (PME) method, and coordinates were recorded every 10 ps, yielding 10,000 frames for analysis.

Structural stability and flexibility were assessed via root-mean-square deviation (RMSD) and root-mean-square fluctuation (RMSF) calculations for the entire complex and the ligand. Global structural compactness was assessed via Radius of Gyration (Rg), while interaction stability was characterized by hydrogen bond occupancy. PCA of backbone atoms was employed to capture dominant collective motions. For free energy estimation, stable segments from the final phase of the simulation were subjected to MM-PBSA analysis with the gmx_MMPBSA tool 42, decomposing the total binding free energy into van der Waals, electrostatic, polar solvation, and nonpolar contributions.

Structural visualization and interaction analyses were performed in PyMOL.

### Quantification and statistical analysis

Statistical analyses were performed using GraphPad Prism. Data distribution was first assessed via Shapiro-Wilk test (3 ≤ n ≤ 50), D'Agostino-Pearson test (50 < n < 1000), or Anderson-Darling test as appropriate. For normally distributed datasets, a two-tailed Student's t-test was used. Otherwise, the non-parametric Mann-Whitney U test was applied. All data are presented as mean ± SEM (or SD as indicated). Statistical significance was defined as P < 0.05 and denoted as follows: * (P < 0.05), ** (P < 0.01), *** (P < 0.001), **** (P < 0.0001) and ns (not significant). Exact n values representing independent biological replicates are provided in each figure legend.

## Results

### HSC aging alters steroid metabolism

**Figure 1:**
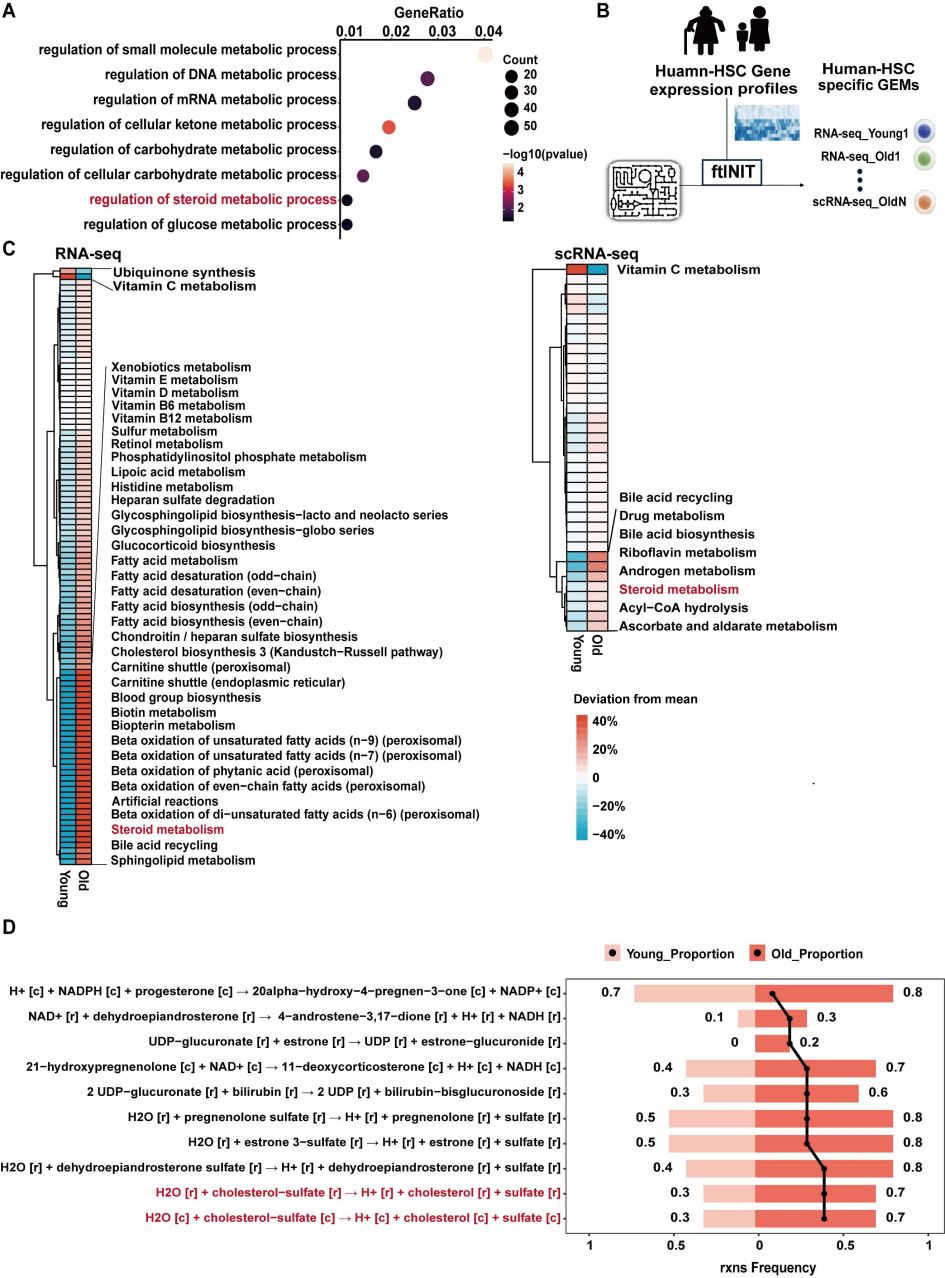
** Steroid metabolism is altered during HSC senescence.** (**A**) Dot plot showing Gene Ontology (GO) metabolic pathway enrichment of the differentially expressed genes (DEGs) during human HSC aging. (**B**) Workflow of GEMs constructed from RNA-seq (Young n = 10, Old n = 10) and scRNA-seq (Young n = 5, Old n = 3) data to prioritize metabolic changes in aging HSCs. (**C**) Comparison of relative subsystem coverage between young and aged human HSCs GEMs, showing only subsystems with top deviation between the two models, based on RNA-seq (left) and scRNA-seq data (right). (**D**) Top 10 metabolic reactions within the steroid metabolism pathway exhibiting the largest increase in reaction ratio in aged compared to young HSCs, based on GEM simulation results.

Previous studies have reported substantial metabolic alterations in aged mouse hematopoietic stem and progenitor cells (HSPCs) 16. To investigate metabolic changes associated with human HSC aging, gene expression profiles associated with human HSC senescence were interrogated, followed by over-representation analysis (ORA) with a focus on metabolic pathways from publicly available datasets 2. Metabolic processes involving small molecules, nucleic acids, ketones, and carbohydrates were significantly enriched, consistent with prior findings 16, 43-47. Notably, biological processes related to steroid metabolism were also significantly enriched (Figure 1A).

Given that changes in transcript abundance do not necessarily reflect metabolic flux, GEMs were constructed to prioritize metabolic alterations *in silico*. GEMs based on bulk RNA-seq and scRNA-seq data from human HSCs revealed marked metabolic differences associated with HSC aging (Figure 1B). Comparative analysis of subsystem coverage revealed significant divergence between young and aged HSCs, particularly in steroid metabolism, across both transcriptomic platforms (Figure 1C).

To further refine these observations, reaction-level flux analysis within the steroid metabolism network was conducted. Several reactions displayed increased predicted activity in aged HSCs, among which CS hydrolysis ranked as one of the most prominently altered nodes (Figure 1D). These data suggest that altered CS metabolism may represent a conserved metabolic feature of HSC aging.

### CS treatment rejuvenated aged HSPC phenotypes

Based on the predicted increase in CS hydrolysis in aged HSCs, the functional consequences of CS supplementation were evaluated *in vivo*. Aged mice were treated with CS to explore this possibility.

**Figure 2:**
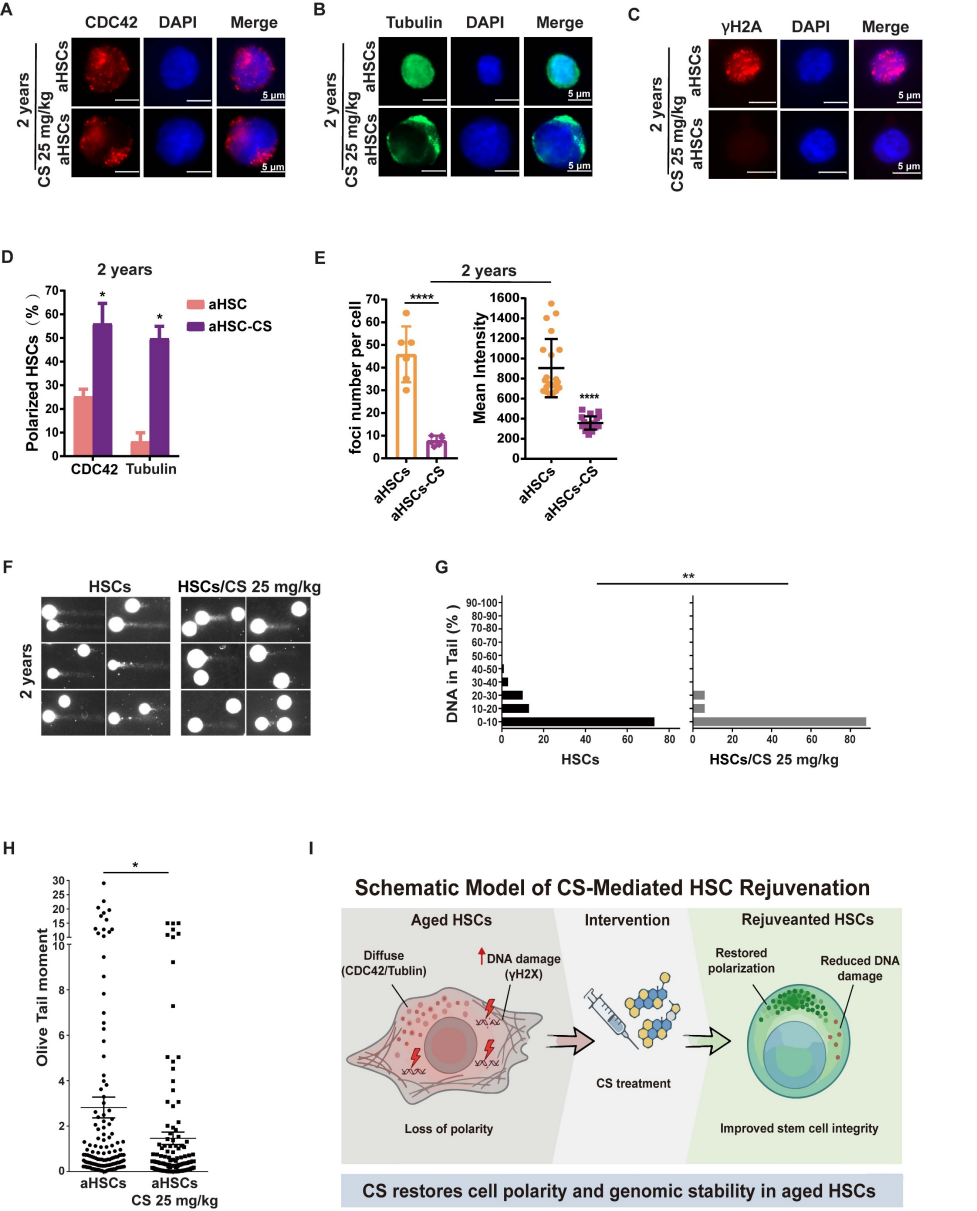
** CS treatment rejuvenates aged HSPC phenotypes.** (**A** and **B**) Immunofluorescence staining of CDC42 (red) (A) and tubulin (B) for a polarity analysis using HSCs isolated from 2-year-old mice treated with CS or vehicle, DAPI (blue) was used to indicate nuclear localization (n = 3, each group). (Scale bar, 5 μm.) (**C**) Immunofluorescence staining of γH2AX (red) in HSCs isolated from 2-year-old mice treated with CS or vehicle for DNA damage analysis; DAPI (blue) for indicating nuclei localization (n = 3, each group). (Scale bar, 5 μm.) (**D**) The percentage of polar cells in each sample from 2-year-old mice was analyzed, and scored as indicated (n = 3, each group). (**E**) For 2-year-old mice, the γH2AX MFI and the number of foci per cell are presented as shown. Four independent replicates were performed. (**F**) Alkaline comet results for HSCs isolated from 2-year-old mice treated with vehicle (F-I) or CS (F-II) as indicated. (**G** and **H**) Percentage of DNA in tail (G-I, II) and (H) olive tail moment of HSCs from 2-year-old mice treated with vehicle or CS as indicated. (**I**) Schematic model of CS-mediated rejuvenation of aged HSCs. Results were represented with the mean ± SEM. *P < 0.05, **P < 0.01, ***P < 0.001 and ****P < 0.0001 were considered as significant difference.

Loss of cell polarity and elevated CDC42 activity are established hallmarks of aged HSCs 48, 49. IF assays on CDC42 and Tubulin (TUBA1A) revealed that the majority of aged HSPCs were re-polarized post CS treatment (Figure 2A-B, D and Figure S1A-B, D). DNA damage burden and repair capacity are important indicators of cellular senescence 50. Aged HSPCs displayed increased γH2AX foci, whereas CS-treated cells showed a marked reduction, with only rare foci detected (Figure 2C, E and Figure S1C, E). Moreover, there was a significant reduction in the HSPC DNA damage burden following CS treatment when compared to untreated cells (Figure 2F-H and Figure S1F-H). Together, these results indicate that CS treatment restores cell polarity and reduces genomic instability in aged HSCs, as summarized in the schematic model in Figure 2I.

CS treatment also resulted in increased proliferation and elevated absolute numbers of HSPCs, including long-term HSCs (LT-HSCs), short-term HSCs (ST-HSCs), and multipotent progenitors (MPPs) (Figure S2A-F). Hematopoietic progenitor cells (HPCs), including Common Myeloid Progenitors (CMPs), Granulocyte-Monocyte Progenitors (GMPs), and Megakaryocyte-Erythroid Progenitors (MEPs) were similarly expanded (Figure S2G-L). Correspondingly, the total white blood cell counts in the peripheral blood (PB), spleen (SPL) and bone marrow (BM) were increased in CS-treated mice (Figure S3A-F). Collectively, these data support a broad enhancement of hematopoietic output following CS treatment.

### CS restores transcriptional and metabolic programs in aged HSCs

**Figure 3:**
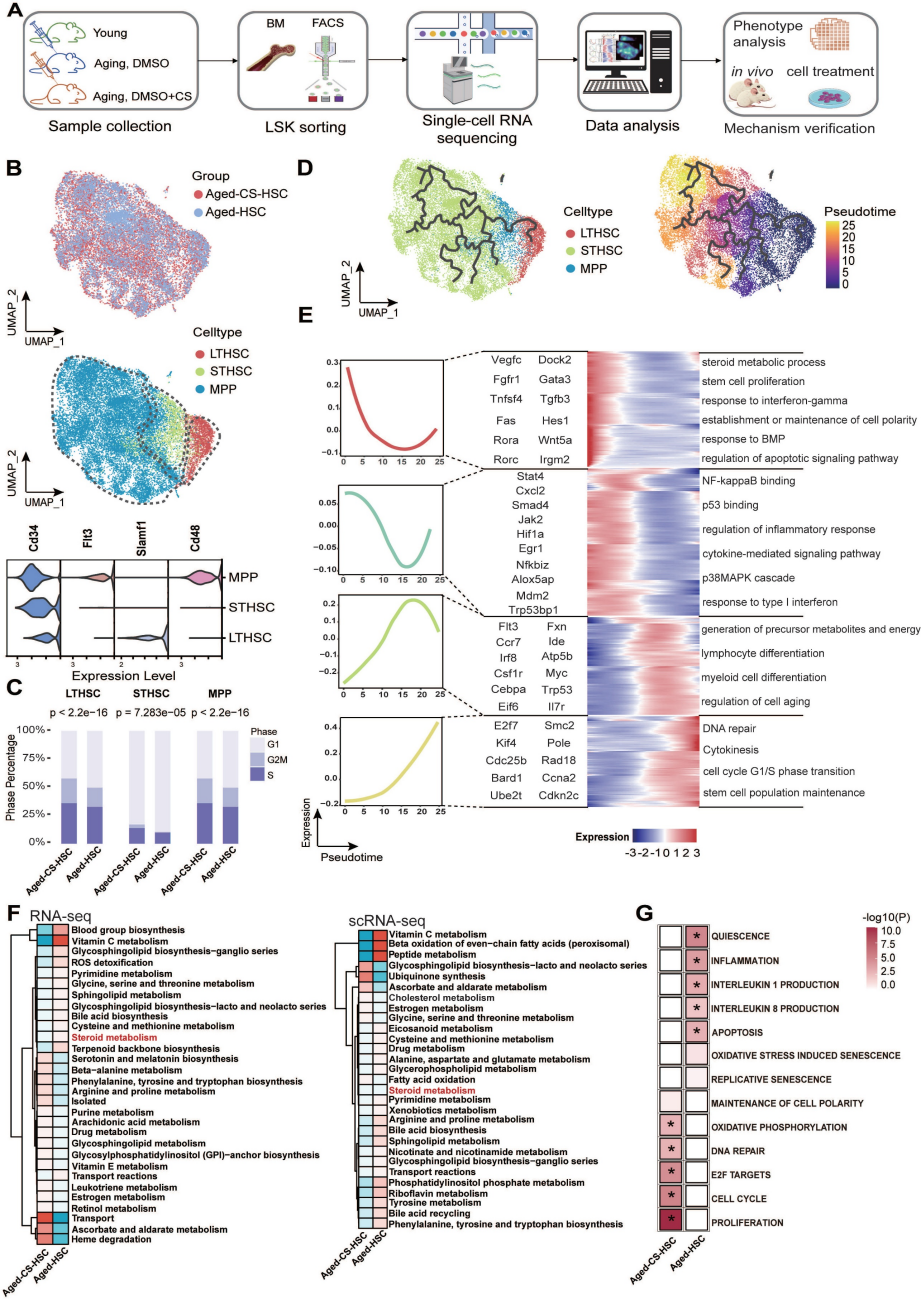
** Single-cell transcriptomic profiling of CS-treated HSCs.** (**A**) Schematic workflow outlining the process of scRNA-seq (n = 3, each group), phenotypic characterization, and mechanistic validation following CS injection in aged mice. (**B**) UMAP visualization of LSK cells from aged and CS-treated aged mice, colored by group (top). UMAP visualization of LT-HSC, ST-HSC, and MPP subpopulations based on their transcriptional profiles (bottom). (**C**) Distribution of cell cycle phases (G1, S, and G2/M) across indicated HSC subsets, quantified via bar charts (chi-square test). (**D**) Pseudotime trajectory of HSCs from combined scRNA-seq data, colored by pseudotime. Pseudotime-ordered analysis of HSCs from the aged and CS-treated aged samples. (**E**) Heatmap showing dynamic changes of highly variable genes along the pseudotime (cataloged hierarchically into four gene modules). Adjusted p value < 0.05 was considered statistically significant for GO enrichment analysis. (**F**) Comparison of relative subsystem coverage between aged and CS-treated aged HSCs GEMs, based on scRNA-seq and RNA-seq data. (**G**) Heatmap of enriched pathways from RNA-seq (n = 3, each group). Differential pathway enrichment between CS-treated and control aged HSCs was determined via GSEA against the MSigDB collection (*P < 0.05, permutation test).

To investigate the molecular mechanisms underlying CS-mediated rejuvenation, HSPCs (LSK, Lin^low^-Sca1^+^-c-Kit^+^) were isolated from aged mice treated with vehicle or CS (Figure 3A) 51. After stringent cell filtration, 19,324 cells were retained for subsequent analyses. We visualized global LSK populations using uniform manifold approximation and projection (UMAP) and identified three distinct HSPC populations corresponding to LT-HSCs, ST-HSCs, and MPPs based on established transcriptional signatures (Figure 3B) 52. CS treatment increased the proportion of cells in S and G2/M phases across compartments, indicating enhanced cell cycle activity (Figure 3C).

Previous studies have demonstrated that hallmarks of HSC aging, including elevated γH2AX foci, loss of polarity (CDC42, tubulin), and chronic inflammation, are progressive with age 48, 53, 54. To further elucidate dynamic changes, a pseudotime trajectory from the combined single-cell dataset of HSCs was reconstructed (Figure 3D). By ordering highly variable genes along this trajectory, we observed an enrichment of genes involved in stem cell proliferation (e.g., *Vegfc*,* Wnt5a*), DNA repair (e.g., *Bard1*,* Ube2t*), and other pathways associated with HSC self-renewal (Figure 3E) 55, 56. Gene set scores calculated over pseudotime demonstrated that pathways linked to the unfolded protein response, cellular senescence, and inflammation were significantly downregulated in the CS-treated group compared to the untreated aged group (Figure S4A). Given the central role of mTOR signaling in stem cell regulation and aging, we specifically evaluated this pathway. Genes in the “Hallmark mTORC1 signaling” pathway were significantly upregulated following CS treatment (Figure S4C and D), suggesting reactivation of anabolic and proliferative programs. Along the pseudotime trajectory, expression of *Mtor*, *Trp53*, and *Myc* increased, while the senescence marker *Cdkn1a (p21)* was markedly reduced in CS-treated cells (Figure S4B).

Bulk RNA-seq analysis corroborated these findings, demonstrating reversal of age-associated transcriptional signatures, activation of DNA repair pathways, and suppression of inflammatory signaling (Figure 3G). Integration of transcriptomic data into GEMs revealed partial restoration of metabolic subsystems, including steroid metabolism, following CS treatment (Figure 3F).

At the protein level, immunofluorescence analysis demonstrated increased expression of total mTOR and phosphorylated mTOR (p-mTOR) in CS-treated aged HSPCs (Figure S4E-H). Given the complex role of mTOR in HSC aging, where hyperactivation drives exhaustion but basal activity is essential for maintenance, we sought to clarify the nature of CS-induced mTOR signaling 57. To assess the magnitude of pathway activation, aged HSCs treated with vehicle, CS, or the potent synthetic mTOR agonist MHY1485 were compared. CS treatment significantly increased p-mTOR, p-S6K, and p-4EBP1 levels relative to vehicle-treated controls, whereas MHY1485 induced substantially higher p-mTOR and p-S6K levels (Figure S5). These results indicate that CS induces a distinct pattern and magnitude of mTOR pathway activation compared with strong pharmacologic stimulation.

In parallel, the DNA repair proteins ATM and PARP1 were upregulated following CS treatment (Figure S4K-N). Interestingly, E2F1 protein levels were reduced despite enrichment of E2F target gene signatures (Figure S4I-J), suggesting post-transcriptional regulation.

### CS treatment clears stress-responsive HSCs

**Figure 4:**
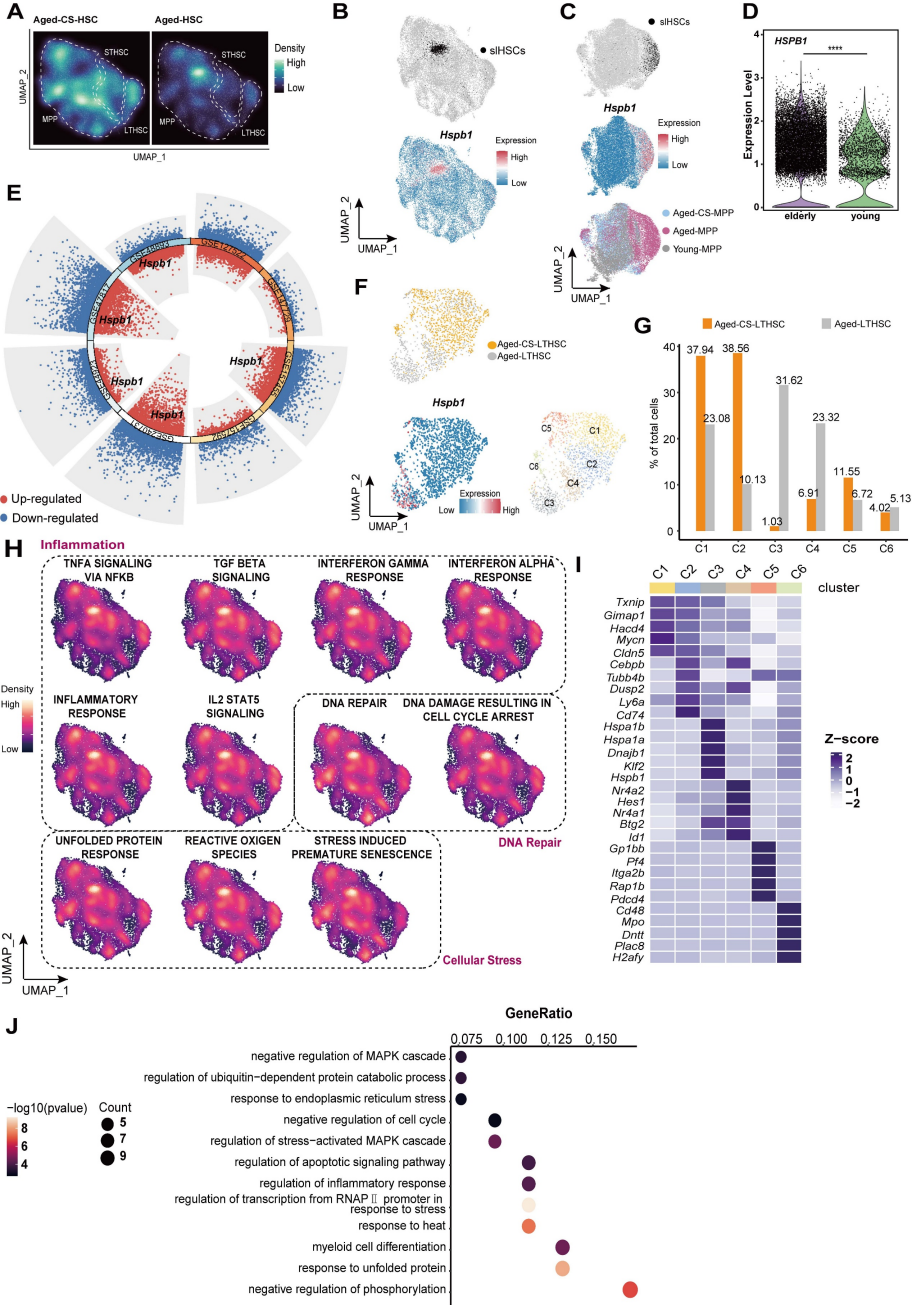
** CS treatment clears senescent-like HSCs.** (**A**) UMAP-based visualization of cell density distributions for CS-treated (left) and untreated (right) aged HSCs, shown as Galaxy plots. (**B**) UMAP plot highlighting a srHSC cluster with high *Hspb1* expression in aged mice. (**C**) UMAP visualization of MPPs from young, aged, and CS-treated aged mice, colored by group (bottom), srHSC across groups (top), and *Hspb1* expression (middle). (**D**)* HSPB1* gene expression of HSCs between elderly and young people by Wilcoxon test, ****P < 0.0001. (**E**) Differential gene expression analysis showing up- and down-regulated genes across eight healthy aged mice versus young mice HSCs. All adjusted p value < 0.01 dots were shown. up-regulated genes are indicated in red, while down-regulated genes are indicated in blue. Genes with an adjusted p value < 0.01 and log2 fold change > 0.263 were considered DEGs. Among these DEGs, *Hspb1* is highlighted, with a total of five instances. (**F**) Cell clusters between aged-CS and aged group were visualized using UMAP (top)**.** UMAP plots showing *Hspb1* gene expression in LTHSCs (bottom left). Identification of 6 subclusters of LTHSCs across all samples (bottom right). (**G**) The proportions of 6 subclusters in HSCs from aged-CS and aged groups. (**H**) UMAP plot of gene signatures for inflammation, DNA repair, and cellular stress pathways in HSCs, generated by UCell. (**I**) Heat map showing the scaled expression of top 5 marker genes in each cell cluster. (**J**) Dot plot showing Gene Ontology (GO) enrichment of highly expressed genes in C3 subcluster.

In the single-cell atlas of HSCs, a distinct cluster of MPPs characterized by high expression of *Hspb1* was identified exclusively in aged mice, but not in CS-treated aged mice (Figure 4A and B). Based on enrichment of inflammatory, stress-response, and DNA repair pathways, this population was designated as stress-responsive HSCs (Figure 4H). This cluster was labeled and projected onto the UMAP dimension reduction space of young, aged, and CS-treated aged MPPs. SrHSCs were uniquely present in the aged group, with hardly detectable presence in young or CS-treated aged mice (Figure 4C). Notably, *Hspb1* shows differential expression in HSCs from normal aged mice and elderly human donors compared to their younger counterparts (Figure 4D and E).

Unsupervised clustering of LT-HSCs further revealed a similar srHSCs, cluster C3 in aged mice, which high expressed *Hspb1* and other genes associated with cellular senescence (Figure 4F and I-J). This cluster was nearly abolished following CS treatment (Figure 4F and G).

Collectively, these findings indicate that CS treatment mitigates aging-associated stress-responsive transcriptional states.

### CS accelerates DNA repair following acute genotoxic stress

**Figure 5:**
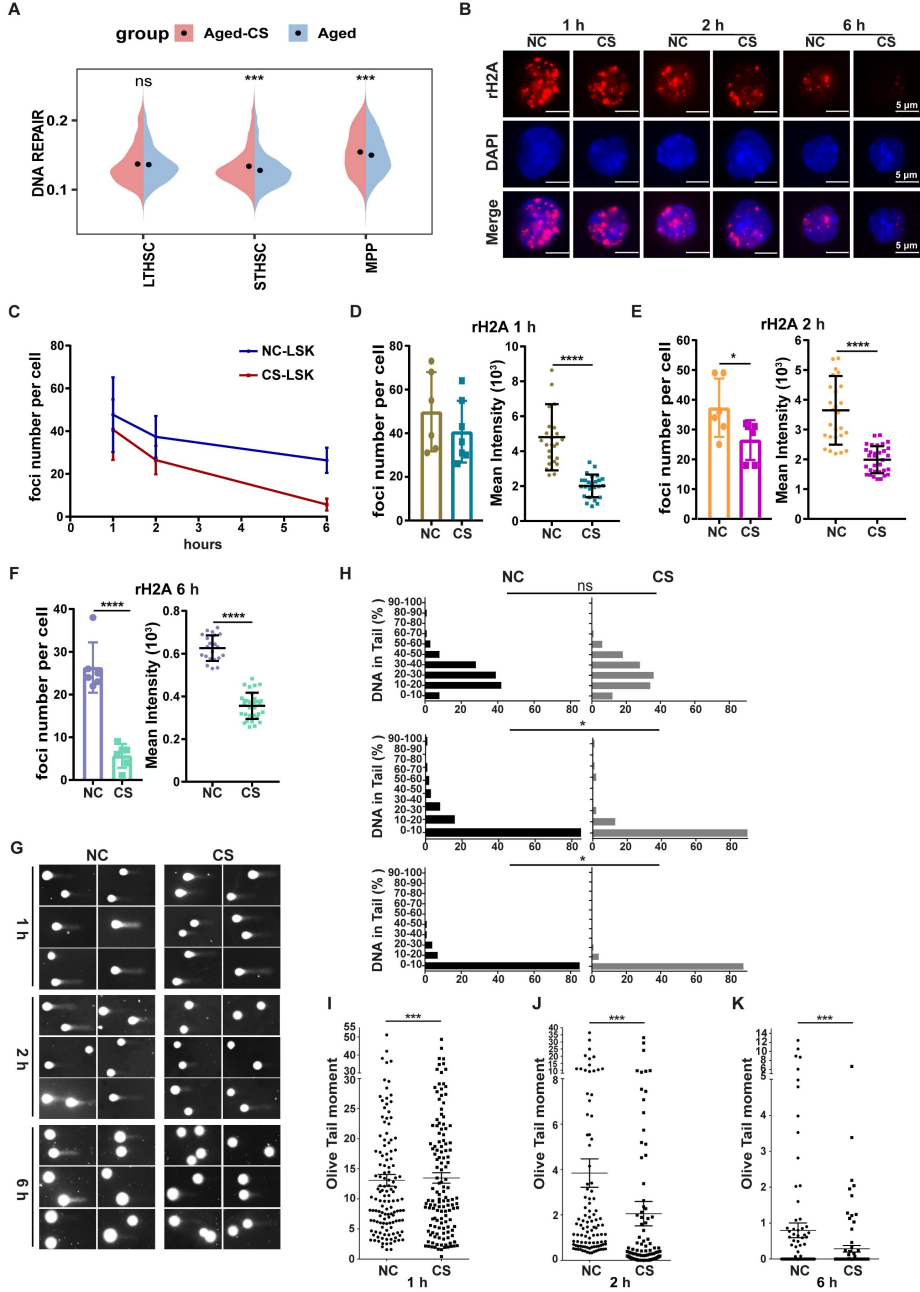
** CS treatment accelerates DNA repair.** (**A**) Violin plots showing the UCell scores of DNA repair pathway in different cell types of aged-CS and aged groups. (two-sided Wilcoxon rank-sum test). (**B**) Following γ-irradiation, γH2AX staining was performed on HSPCs isolated from DMSO- or CS-treated mice at specific intervals (red). DAPI (blue) was to indicate nuclei (n = 3, each group). (Scale bar, 5 μm.) (**C**-**F**) The average number of γH2AX foci (C) and the average MFI intensity value in each sample at 1 (D), 2 (E) and 6 h (F) were summarized and indicated. All the data were summarized from at least three individual mice and for a total of no less than 80 cells. (**G**-**K**) The post irradiation alkaline comet results for HSCs from DMSO or CS treated at 1, 2, 6 h (G). Summary of the percentage of DNA in tails (H) and the olive distance (I, J and K) for HSCs from experimental and control mice at each time period (n = 3, each group). Error bars indicate the mean ± SEM. Comparisons between groups were performed via the Mann-Whitney test. *P < 0.05, **P < 0.01, ***P < 0.001, and ****P < 0.0001 were considered statistically significant, whereas 'ns' indicates no significance (D-F and H-K).

The data contained herein demonstrated that CS reversed the amount of DNA damage level in HSPCs from aged mice (Figure 2G-H), which suggests that CS influences DNA damage repair processes. The transcriptome sequencing analysis also revealed that there was an expression increase of genes involved in DNA repair pathways following CS treatment (Figure 5A). To further demonstrate the role of CS in DNA damage repair, an acute DNA damage mouse model was constructed by use with low-dose gamma-ray irradiation. Post irradiation mice were sacrificed at different time points and HSPCs were isolated for an analysis to identify DNA damage lesions. At the examined time points, the DNA repair process was significantly faster, reflected by fewer γH2AX foci within HSCs from the CS treated group (Figure 5B, D-F). The rate of γH2AX foci decline was greater in the CS treatment group than the decline in the control group mice, which indicates that CS treatment also accelerated the DNA repair speed (Figure 5C). To assess the accumulation of DNA damage in irradiated HSCs, a quantitative assessment of DNA damage was performed. The comet assay demonstrated that the accumulation of DNA damage burden in HSPCs from CS treated mice was significantly lower (Figure 5H-K). These results indicate that CS treatment is associated with accelerated DNA repair following genotoxic stress.

### CS-induced HSCs Proliferation via RORA activation

**Figure 6:**
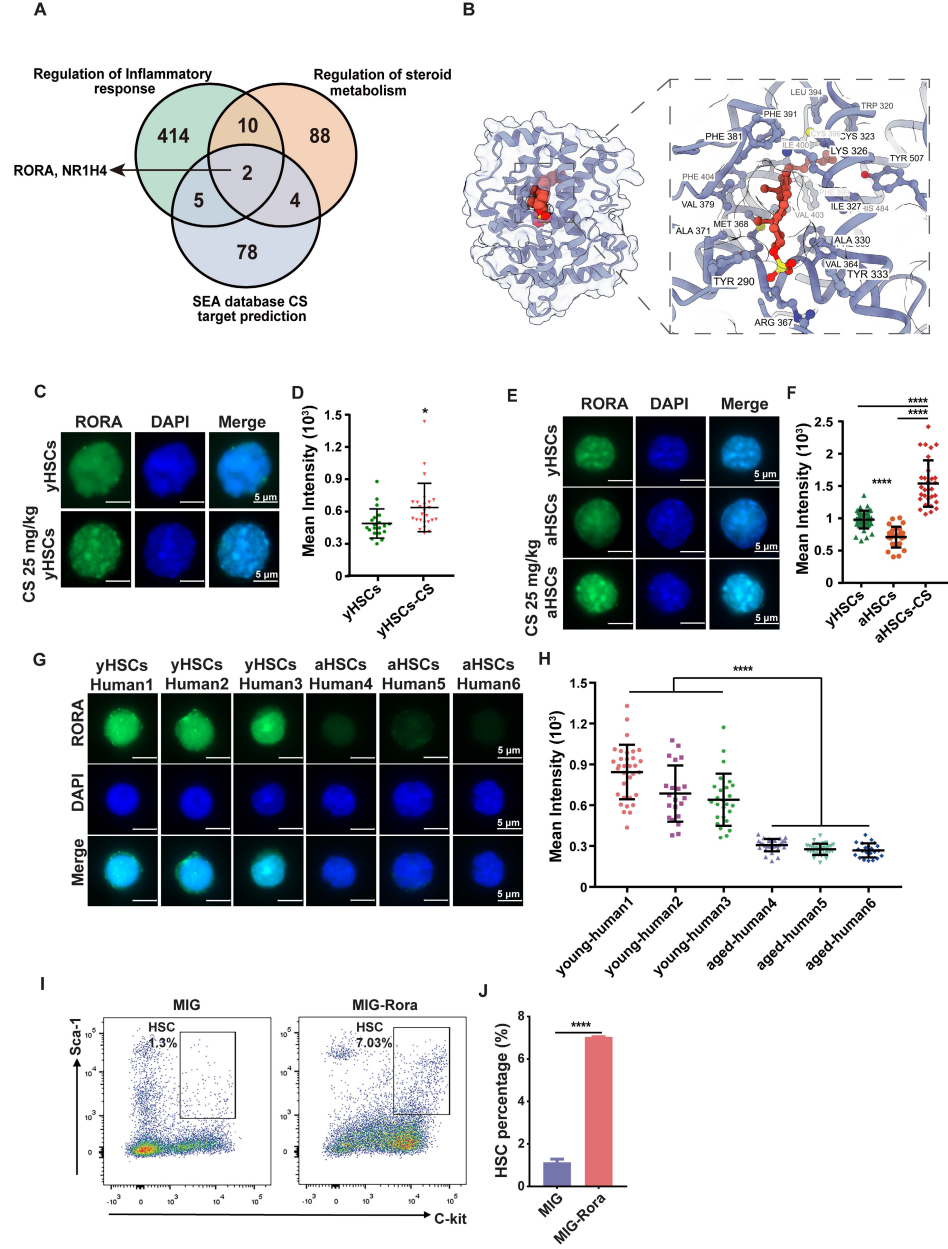
** CS treatment promotes HSC proliferation via RORA activation.** (**A**) Venn plot showing the number of genes shared among gene sets related to inflammation, steroid metabolism, and SEA-predicted targets of CS, identifying *Rora* and *Nr1h4* as common nodes. (**B**) Molecular dynamics simulation snapshot of the RORA-CS complex at the final time point. The RORA protein is visualized in both atomic surface and cartoon representations, while the bound CS molecule is highlighted as red spheres, illustrating its stable positioning within the LBD. (**C**) Immunofluorescence staining of RORA in HSCs from young mice, treated with or without CS, as indicated (n = 3, each group). (Scale bar, 5 μm.) (**D**) The MFI in each individual cell was analyzed and the dots indicate the individual counted cells (n = 3, each group).(**E**)Immunofluorescence staining of RORA (green) in young HSCs (E-I), DAPI (E-II), and merge (E-III); aged HSCs (E-IV), DAPI (E-V), and merge (E-VI); aged HSCs treated with CS (E-VII), DAPI (E-VIII), and merge (E-IX) (n = 3, each group). (Scale bar, 5 μm.) (**F**) MFI of RORA in each individual cell, the total cell counts were as indicated. (**G**) RORA protein in human HSCs (CD34+ cells) (young and aged, n = 3, each group). (Scale bar, 5 μm.) (**H**) The MFI in each individual cell was analyzed and indicated using dots (n = 3, each group). (**I** and** J**) FACS diagram (I) showing the number and percentage (J) of HSCs in BM from recipients that received wild-type (WT) BM cells transduced with MIG or MIG-*Rora* retroviruses four weeks post BMT (n = 3, each group). Results were represented with the mean ± SEM. *P < 0.05, **P < 0.01, ***P < 0.001 and ****P < 0.0001 were considered as significant difference (Mann-Whitney test).

To identify functional targets mediating the pro-regenerative effects of CS, SEA-predicted targets (Table S3) were intersected with pathways associated with inflammation and steroid metabolism. This analysis revealed RORA and NR1H4 as overlapping candidates (Figure 6A). Given the established role of RORA in regulating HSC function and maintenance, subsequent analyses focused on this receptor 58. In contrast, NR1H4 primarily associated with bile acid metabolism, has limited reported relevance in HSC biology.

We next examined whether CS directly binds to RORA using molecular docking and molecular dynamics simulations. Docking predicted a stable binding pose of CS within the LBD of RORA (Figure 6B). CS formed more frequent hydrogen bonds and exhibited a lower binding free energy than 4ACD8, a sterol-like RORA ligand co-crystallized in the RORα structure (PDB: 4S15), which was used as a positive control (Figure S6E-H, Figure S7E-G). Although the RMSD and RMSF values of the CS-RORA complex were slightly higher, they remained within the stable range of 1-3 Å, indicating acceptable conformational stability (Figure S6A-D, Figure S7A-D, Table S4). These findings are consistent with earlier crystallographic studies identifying CS as a physiological ligand of RORA 59, 60.

Immunofluorescence staining revealed that RORA protein was downregulated in aged mice and human HSCs compared to young controls (Figure 6C-H). Notably, CS treatment significantly enhanced RORA expression in HSCs from young mice compared to untreated controls (Figure 6C-D), and restored RORA expression in HSCs from aged mice to youthful levels (Figure 6E-F). We further tested whether RORA is functionally sufficient to drive HSC expansion. *Rora* was overexpressed in HSPCs via retroviral transduction, followed by BMT. HSPCs were analyzed four weeks post BMT and a greater number of HSPCs were detected in the recipients of the cells with high *Rora* expression compared to the vector group recipients (Figure 6I-J).

Conversely, conditional deletion of *Rora* in young HSCs resulted in premature aging phenotypes, including loss of polarity and increased DNA damage by employing a lineage-specific knockout model (*Rora^fl/fl^ Mx1-Cre*) (Figure S8). These findings suggest that RORA is essential for maintaining the core homeostatic hallmarks of youthful HSCs. Collectively, these findings support the CS-RORA axis as a key mechanistic contributor to CS-mediated rejuvenation of aged HSCs.

## Discussion

While significant progress has been made in characterizing the transcriptional and epigenetic changes accompanying HSC aging, effective and safe strategies to reverse these phenotypes remain limited. Over the past decade, transcriptomic technologies have generated vast public datasets across developmental, aging, and disease contexts, yet strategies to systematically integrate and functionally mine these resources remain underdeveloped. To address this gap, we established a human data-driven, metabolism-centered strategy to systematically identify endogenous compounds with rejuvenating potential in aged HSCs. By constructing GEMs from transcriptomic profiles of purified human HSCs, we captured aging-associated metabolic alterations and prioritized naturally depleted metabolites that may underlie functional decline (Figure 1). This approach bridges systems biology with clinically relevant compound discovery and is guided by the broadly accepted principle of restoring what is lost, a concept rooted in both traditional medicine and modern metabolic therapy frameworks 9,10.

Herein, we identified CS, a naturally occurring sterol conjugate, as a key metabolic regulator of HSC aging. CS supplementation in aged mice reversed multiple hallmarks of aging across transcriptional, metabolic, and functional dimensions (Figure 2-3 and Figure S1-4), highlighting its potential as a safe and physiologically compatible therapeutic candidate. At the single-cell level, CS eliminated a transcriptionally defined population of srHSCs marked by elevated *Hspb1* (Figure 4), a change that was also observed in independent aging datasets, suggesting conserved relevance. Due to the role CS plays in accelerating DNA damage repair (Figure 5), CS has potential applications for the reduction of DNA damage following irradiation therapy and accidental radiation expose.

Mechanistically, our data support a model in which RORA functions as a key mediator downstream of CS. This link is supported by prior structural studies that solved the RORA ligand-binding domain and identified cholesterol sulfate as a natural ligand 59, 60. In our study, CS activated a RORA-associated transcriptional program, and enforced *Rora* expression was sufficient to reproduce a major component of the CS phenotype, namely HSC expansion (Figure 6). Importantly, to begin addressing necessity, we leveraged an inducible conditional knockout model and found that *Rora* deletion in young mice elicited premature aging-like defects in HSC polarity and genomic integrity (Figure S8), two mechanistic features strongly implicated by our CS response data. In conclusion, RORA was identified as a pivotal functional mediator that sustains juvenile HSC homeostasis and orchestrates the primary effects of CS. Furthermore, mTOR signaling influences HSC senescence in a sophisticated, context-specific manner. While mTOR activity is increased in old HSCs, more recent studies suggest that subsets of aged HSCs exhibit suppressed mTORC1 activity 53, 57. Our transcriptomic and protein-level analyses revealed that CS treatment modestly reactivated mTOR signaling (Figure S5). This pattern supports a model in which CS promotes a restrained, physiologically balanced reactivation of the mTOR network, potentially sufficient to support repair and proliferative competence without forcing the pathway into a hyperactivated exhaustion-prone state. Additionally, we observed that CS treatment enriched E2F target gene expression while reducing E2F1 protein levels. This apparent paradox may reflect post-transcriptional feedback or regulatory cross-talk. Prior work has shown that RORA can bind and repress E2F1 transcriptional activity, suggesting that RORA activation may help balance proliferative signaling without triggering aberrant hyperproliferation 61.

We also acknowledge several limitations that will need to be addressed in future work. Although we observed improved proliferation, reduced DNA damage, and enhanced hematopoietic output, competitive transplantation or serial transplantation assays are lacking. Thus, the extent to which CS restores long-term self-renewal and clonal durability remains to be determined. The computational discovery pipeline is based on human datasets. However, functional testing was primarily conducted in mice. Direct *ex vivo* validation in primary human CD34^+^HSPCs, such as CS-responsive transcriptomic profiling, will be an important next step to enhance translational relevance. Given that CS is a cholesterol-derived sterol conjugate, potential off-target effects through other sterol-sensing nuclear receptors should also be considered. Notably, LXR activation has been reported to induce the sterol sulfotransferase SULT2B1b 62, and SULT2B1b-mediated formation of sulfated sterols can suppress LXR/SREBP-1c signaling 63. Consistently, LXR-linked cholesterol efflux via ABCA1/ABCG1 and HDL restrains HSC proliferation *in vivo* 64, which contrasts with the robust HSC expansion observed with CS supplementation in aged mice. These considerations argue against LXR activation being the dominant driver of the CS phenotype in our setting. Nevertheless, we cannot formally exclude auxiliary contributions from LXR- or FXR-associated mechanisms, particularly via indirect or systemic effects. Therefore, while our data support RORA as a key mediator of CS responsiveness, future studies combining CS administration with receptor-specific genetic ablation or pharmacologic perturbation will be required to rigorously dissect the relative contributions of RORA versus other sterol-sensing pathways.

Our identification of CS adds to the expanding repertoire of metabolites reported to modulate aging-associated stem cell phenotypes. Uridine was reported to activate FoxO signaling, enhance self-renewal, and suppress inflammation in aged HSCs 16. In parallel, α-ketoglutarate has been shown to extend adult C. elegans lifespan by binding and inhibiting ATP synthase subunit β, leading to reduced ATP production and oxygen consumption, increased autophagy, and a TOR-dependent longevity program 65. Distinct from these nucleoside- and mitochondrial energy-linked mechanisms, our study supports a model in which CS, as a sterol-derived signaling metabolite, engages the nuclear receptor RORA as a key mediator to restore HSC polarity and genomic integrity while suppressing inflammatory programs. Together, these findings highlight that metabolic interventions may influence aging through complementary nodes, ranging from transcription factor networks to mitochondrial energy metabolism and autophagy.

## Conclusions

In summary, this study identifies CS as a critical endogenous metabolite that declines during HSC aging. CS supplementation effectively rejuvenates aged HSCs by restoring cell polarity, mitigating genomic instability, and enhancing hematopoietic output. Mechanistically, the CS-RORA axis functions as a key mediator of this metabolic rejuvenation. Given the endogenous nature and safety of CS, these results highlight the potential of CS replenishment as a promising and translational metabolic strategy to reverse age-related hematopoietic decline.

## Supplementary Material

Supplementary figures and tables.

## Data Availability

The transcriptomic datasets utilized in this research are publicly accessible via the Gene Expression Omnibus (GEO) repository (https://www.ncbi.nlm.nih.gov/geo/). Specific sample details, including group designations and accession IDs, are compiled in Table S5. The primary RNA-seq and scRNA-seq files generated during this work have been archived at the Genome Sequence Archive (GSA) under the identifiers CRA022372 and CRA021800, respectively. Computational scripts are hosted on GitHub at https://github.com/ziweihf/CS-Replenishment-Rejuvenates-Aged-HSC. All other data necessary to support the conclusions of this paper are provided within the manuscript or its supplementary files. Further inquiries regarding data reanalysis should be directed to the corresponding author.

## Abbreviations

scRNA-seq: single-cell RNA sequencing
CS: cholesterol sulfate
BM: Bone marrow
HSPC: Hematopoietic Stem and Progenitor Cell
HSC: Hematopoietic stem cell
PCA: Principal component analysis
ORA: over-representation analysis
GSEA: Gene Set Enrichment Analysis
CML: chronic myeloid leukemia
MDS: myelodysplastic syndromes
GEM: Genome-scale Metabolic
TPM: Transcripts Per Million
CCA: Canonical Correlation Analysis
LT-HSC: Long-Term HSC
ST-HSC: Short-Term HSC
RMSF: root-mean-square fluctuation
MPP: Multi-Potent Progenitors
UMAP: uniform manifold approximation and projection
CMP: Common Myeloid Progenitors
GMP: granulocyte-monocyte progenitor
LBD: canonical ligand-binding domain
srHSCs: stress-responsive HSCs
PME: Particle Mesh Ewald
RMSD: root-mean-square deviation
NVT: constant number, volume, and temperature
MEP: Megakaryocyte-Erythroid Progenitor
NPT: constant number, pressure, and temperature
BMT: bone marrow transplantation
LXR: Liver X Receptor
WT: Wild-type
