# Supplementary material for: Cholesterol sulfate replenishment rejuvenates aged hematopoietic stem cell phenomes
Source: Theranostics. 2026 Mar 17;16(10):5501–17. doi: 10.7150/thno.126134 (PMC13080657; doi:10.7150/thno.126134)
Supplement: Supplementary file 1 — Supplementary figures and tables. [file thnov16p5501s1.zip › Supplementary Materials.pdf]

This PDF file includes:

Supplementary Figs. Figure S1-8.

Other Supplementary Materials for this manuscript include the following:

Table S1. Detailed antibody panels and surface marker definitions.
Table S2. Primary and Secondary Antibodies Targeting Key Proteins.
Table S3. SEA-predicted targets of CS, related to Figure 6.
Table S4. Quantitative metrics from Molecular Dynamics (MD) simulations of RORA complexes
Table S5. Detailed information of all datasets used in this study.

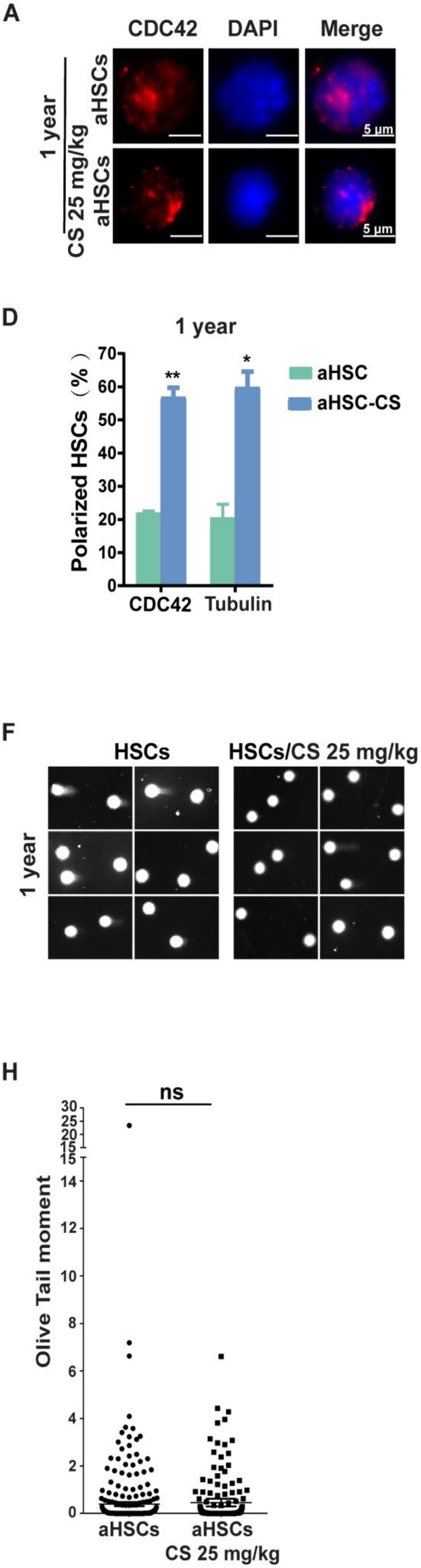

A
CDC42
DAPI
Merge
1 year
aHSCs
CS 25 mg/kg aHSCs
5 μm
B
Tubulin
DAPI
Merge
1 year
aHSCs
CS 25 mg/kg aHSCs
5 μm
C
γH2A
DAPI
Merge
1 year
aHSCs
CS 25 mg/kg aHSCs
5 μm
D
1 year
Polarized HSCs （%）
aHSC
aHSC-CS
CDC42
Tubulin
**
*
E
1 year
foci number per cell
Mean Intensity
****
aHSCs
aHSCs-CS
F
HSCs
HSCs/CS 25 mg/kg
1 year
G
ns
DNA in Tail (%)
90-100
80-90
70-80
60-70
50-60
40-50
30-40
20-30
10-20
0-10
HSCs
HSCs/CS 25 mg/kg
H
ns
Olive Tail moment
aHSCs
aHSCs
CS 25 mg/kg

Figure S1. CS treatment Rejuvenated Aged HSPC phenotypes in 1-year-old mice. (**A** and **B**) Immunofluorescence staining of CDC42 (red) (A) and tubulin (B) for a polarity analysis using HSCs isolated from 1-year-old mice treated with CS or vehicle, DAPI (blue) was used for indicating nuclei localization (n = 3, each group). (Scale bar, 5 μm.) (**C**) Immunofluorescence staining of γH2AX (red) in HSCs isolated from 1-year-old mice treated with CS or vehicle for DNA repairing analysis; DAPI (blue) for indicating nuclei localization (n = 3, each group). (Scale bar, 5 μm.) (**D**) The percentage of polar cells in each sample from 1 year was analyzed, and scored as indicated (n = 3, each group). (**E**) The MFI of γH2AX in each individual cell and foci numbers in the individual cells from 1 year were displayed as indicated, four independent replicates were performed. (**F**) Alkaline comet results for HSCs isolated from 1-year-old mice treated with vehicle (F-I) or CS (F-II) as indicated. (**G** and **H**) Percentage of DNA in tail (G-I, II) and (H) olive tail moment of HSCs from 1-year-old mice treated with vehicle or CS as indicated. Results were represented with the mean ± SEM. *P < 0.05, **P < 0.01, ***P < 0.01 and ****P < 0.0001 were considered as significant difference, whereas 'ns' indicates no significance.

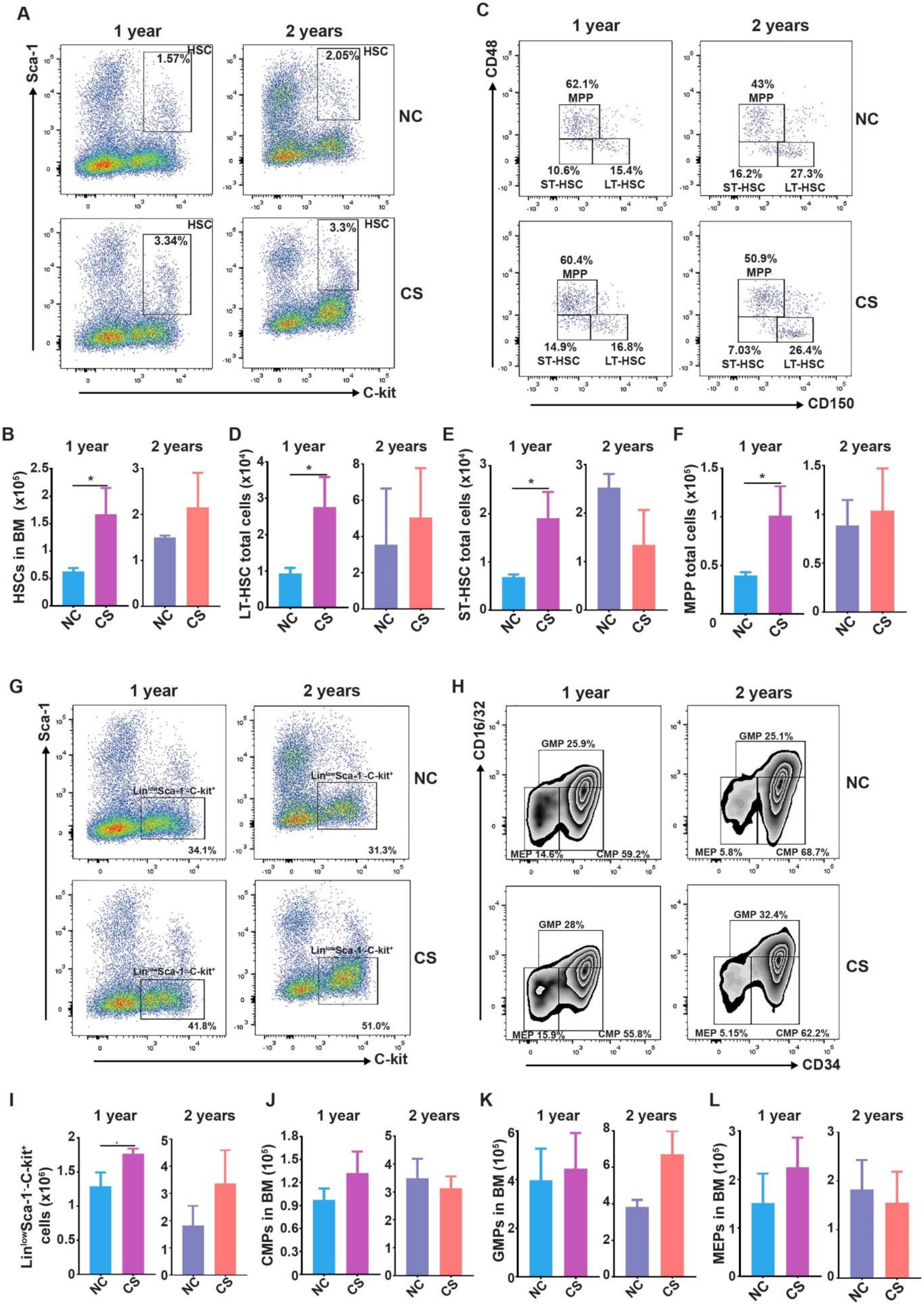

A
1 year
2 years
Sca-1
C-kit
HSC
1.57%
2.05%
3.34%
3.3%
NC
CS
B
HSCs in BM (x10⁵)
C
CD48
CD150
62.1% MPP
10.6% ST-HSC
15.4% LT-HSC
43% MPP
16.2% ST-HSC
27.3% LT-HSC
60.4% MPP
14.9% ST-HSC
16.8% LT-HSC
50.9% MPP
7.03% ST-HSC
26.4% LT-HSC
D
LT-HSC total cells (x10⁴)
E
ST-HSC total cells (x10⁴)
F
MPP total cells (x10⁵)
G
Lin^low Sca-1⁻C-kit⁺
34.1%
31.3%
41.8%
51.0%
H
CD16/32
CD34
GMP 25.9%
MEP 14.6%
CMP 59.2%
GMP 25.1%
MEP 5.8%
CMP 68.7%
GMP 28%
MEP 15.9%
CMP 55.8%
GMP 32.4%
MEP 5.15%
CMP 62.2%
I
Lin^low Sca-1⁻C-kit⁺ cells (x10⁶)
J
CMPs in BM (10⁵)
K
GMPs in BM (10⁵)
L
MEPs in BM (10⁵)

Figure S2. The biological effects on HSPCs in aged mice following RORA activation. (**A**-**C**) A FACS diagram for HSCs (number and percentages) in BM the groups indicated: percentage of HSCs in 1- or 2-year-old mice treated with DMSO (negative control, NC) or CS (A). The total number of HSCs in 1- or 2-year-old mice treated with DMSO or CS as indicated (B). FACS diagram of LT-HSCs, ST-HSCs, and MPPs as well as their percentages in 1- or 2-year-old mice treated with DMSO or CS as indicated (C) (n = 3, each group). (**D**-**F**) The total number of LT-HSCs (D), ST-HSCs (E), and MPPs (F) in 1- or 2-year-old mice treated with DMSO or CS as indicated (n = 3, each group). (**G**-**I**) FACS diagrams and percentages of HPCs in the BM of the indicated groups: The percentage of HSCs in 1- or 2-year-old mice treated with DMSO or CS (G). FACS diagram detailing the percentages for CMPs, GMPs, and MEPs from 1- or 2-year-old mice treated with DMSO or CS as indicated (H). The total number of HPCs in 1- or 2-year-old mice treated with PBS or CS as indicated (I) (n = 3, each group). (**J**-**L**) The total number of CMPs (J), GMPs (K), and MEPs (L) in 1- or 2-year-old mice treated with DMSO or CS as indicated. Three independent experiments were conducted, with n = 3 mice per group in each session. Data are visualized as bar graphs, with error bars representing mean ± SEM. Statistical differences were assessed through the Mann-Whitney U test, with $P < 0.05$ considered significant (*) (B-F and I-L).

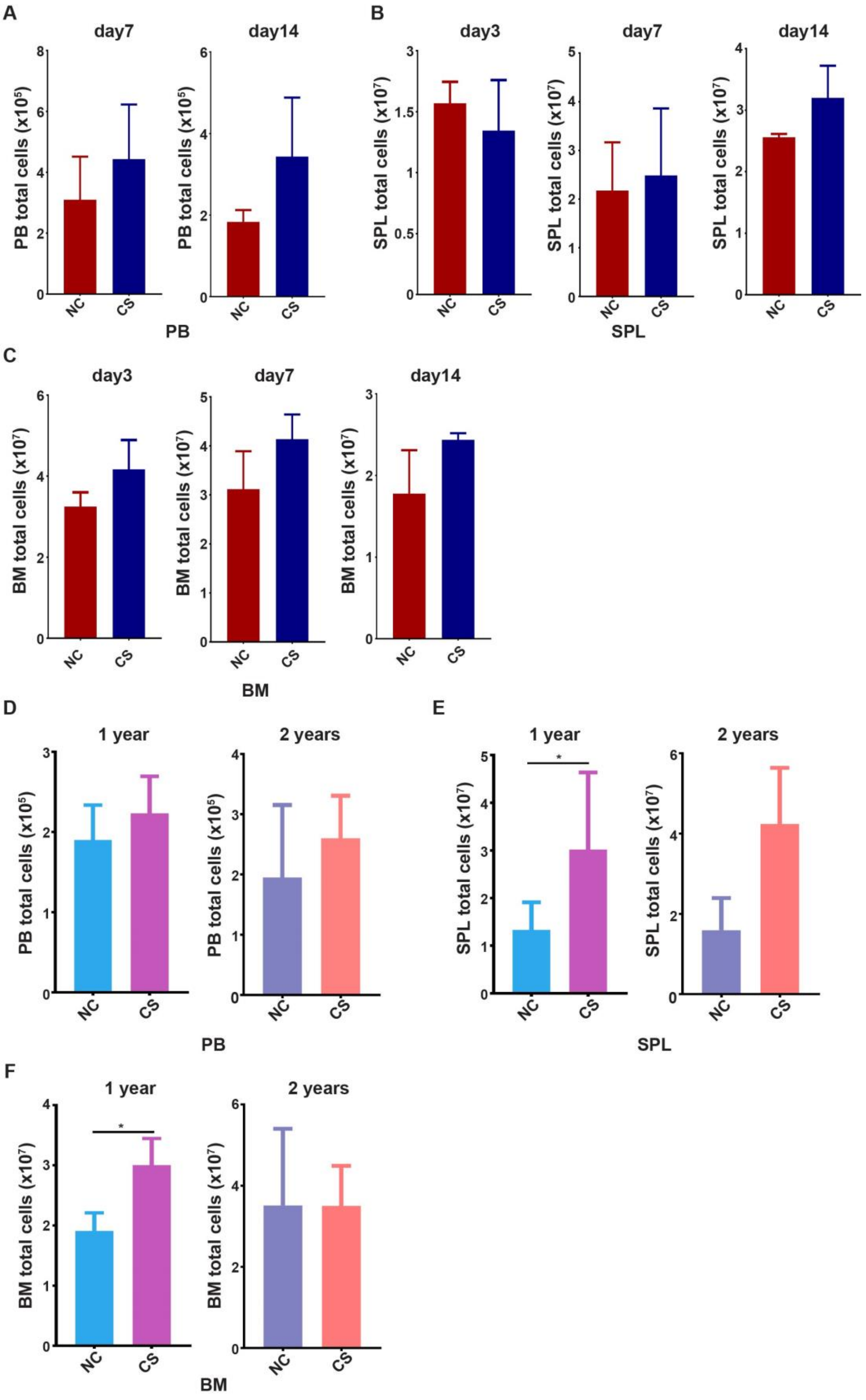

A
day7
day14
PB total cells (x10⁵)
NC
CS
PB
B
day3
day7
day14
SPL total cells (x10⁷)
NC
CS
SPL
C
day3
day7
day14
BM total cells (x10⁷)
NC
CS
BM
D
1 year
2 years
PB total cells (x10⁵)
NC
CS
PB
E
1 year
2 years
*
SPL total cells (x10⁷)
NC
CS
SPL
F
1 year
2 years
*
BM total cells (x10⁷)
NC
CS
BM

Figure S3. The effects of RORA on the number of whole blood cells in young and aged mice. (**A**-**F**) Young (10 weeks) and old (1year and 2years) mice were intraperitoneally injected DMSO (negative control, n = 3) or CS (25 mg/kg, n = 3), twice a day for three, seven, and 14 days for young mice; old mice is only treated for 14 days Peripheral blood (PB) (A, D), spleen (SPL) (B, E), and bone marrow (BM) (C, F) were collected from mice and analyzed individually. Total cells from whole spleen or BM (tibias and femurs) were counted as indicated. Data are visualized as bar graphs, with error bars representing mean ± SEM. Statistical differences were assessed through the Mann-Whitney U test, with $P < 0.05$ considered significant (*).

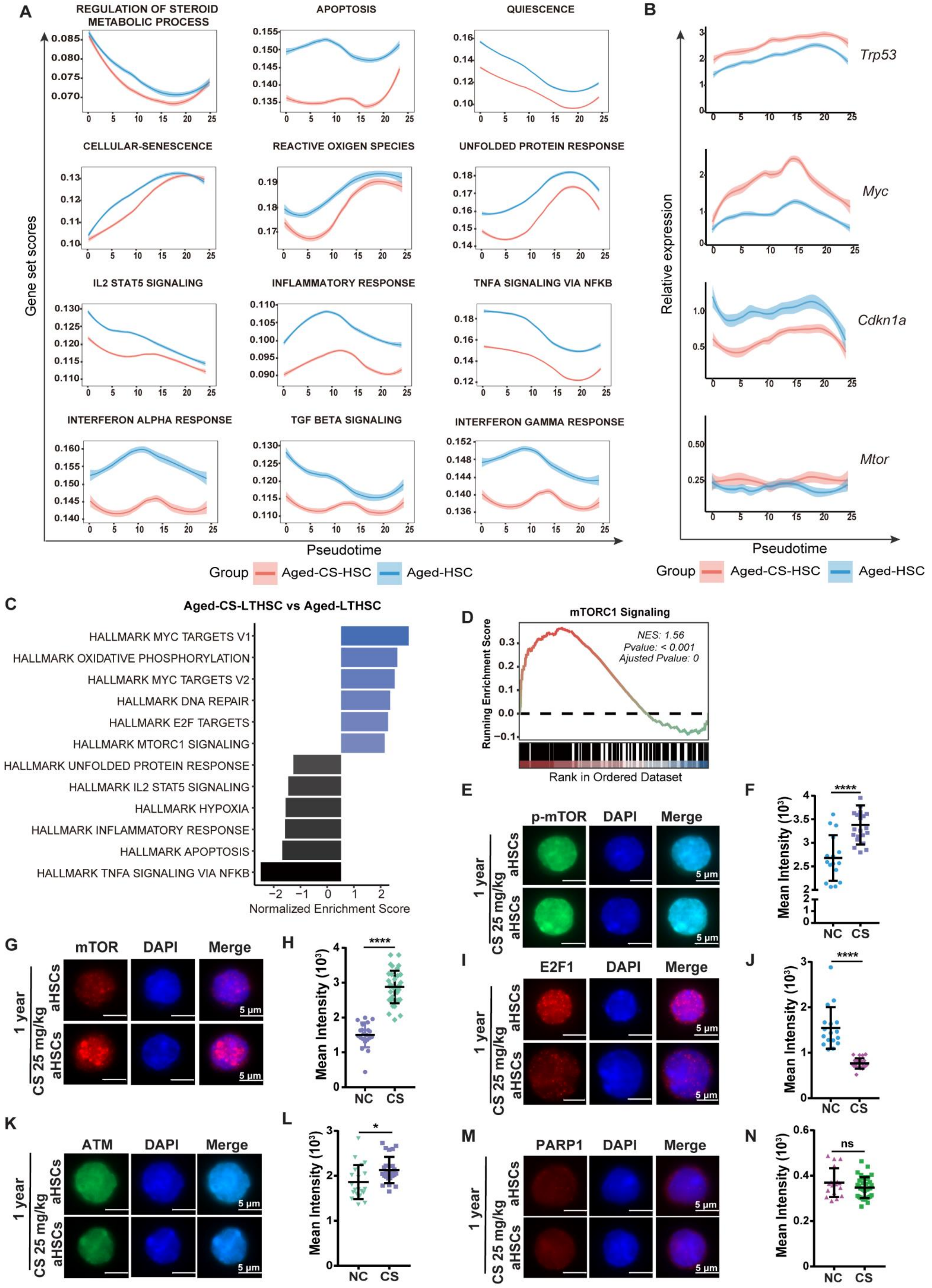

A
REGULATION OF STEROID METABOLIC PROCESS
APOPTOSIS
QUIESCENCE
CELLULAR-SENESCENCE
REACTIVE OXIGEN SPECIES
UNFOLDED PROTEIN RESPONSE
IL2 STAT5 SIGNALING
INFLAMMATORY RESPONSE
TNFA SIGNALING VIA NFKB
INTERFERON ALPHA RESPONSE
TGF BETA SIGNALING
INTERFERON GAMMA RESPONSE
Gene set scores
Pseudotime
Group Aged-CS-HSC Aged-HSC
B
Trp53
Myc
Cdkn1a
Mtor
Relative expression
Pseudotime
Group Aged-CS-HSC Aged-HSC
C
Aged-CS-LTHSC vs Aged-LTHSC
HALLMARK MYC TARGETS V1
HALLMARK OXIDATIVE PHOSPHORYLATION
HALLMARK MYC TARGETS V2
HALLMARK DNA REPAIR
HALLMARK E2F TARGETS
HALLMARK MTORC1 SIGNALING
HALLMARK UNFOLDED PROTEIN RESPONSE
HALLMARK IL2 STAT5 SIGNALING
HALLMARK HYPOXIA
HALLMARK INFLAMMATORY RESPONSE
HALLMARK APOPTOSIS
HALLMARK TNFA SIGNALING VIA NFKB
Normalized Enrichment Score
D
mTORC1 Signaling
Running Enrichment Score
NES: 1.56
Pvalue: < 0.001
Ajusted Pvalue: 0
Rank in Ordered Dataset
E
p-mTOR DAPI Merge
1 year
aHSCs
CS 25 mg/kg aHSCs
5 μm
F
Mean Intensity (10³)
****
NC CS
G
mTOR DAPI Merge
1 year
aHSCs
CS 25 mg/kg aHSCs
5 μm
H
Mean Intensity (10³)
****
NC CS
I
E2F1 DAPI Merge
1 year
aHSCs
CS 25 mg/kg aHSCs
5 μm
J
Mean Intensity (10³)
****
NC CS
K
ATM DAPI Merge
1 year
aHSCs
CS 25 mg/kg aHSCs
5 μm
L
Mean Intensity (10³)
*
NC CS
M
PARP1 DAPI Merge
1 year
aHSCs
CS 25 mg/kg aHSCs
5 μm
N
Mean Intensity (10³)
ns
NC CS

Figure S4. RORA Activation Ameliorates Age-Related Metabolic Dysregulation and Senescence in HSCs. (**A**) Two-dimensional plots showing the dynamic expression of pathway scores along with the pseudotime in aged (blue) and CS-treated aged (red) groups. (**B**) Dynamic expression of representative genes along the pseudotime trajectory. (**C**) Distribution of Normalized Enrichment Scores (NES) for the most significantly altered GSEA pathways in aged LTHSCs after CS treatment (thresholds: |NES| > 1, P < 0.05). (**D**) GSEA revealed that genes involved in mTOR signaling were significantly increased and enriched in the HSCs post CS treatment. (**E**-**N**) Immunofluorescence staining of p-mTOR (E, F) (green), mTOR (G, H) (red), E2F1 (I, J) (red), ATM (K, L) (green) and PARP1 (M, N) (red) of HSPCs isolated from 1 year-old mice treated with vehicle or CS (n = 3, each group). DAPI (blue) was used to indicate nuclei localization. The total number of counted cells was indicated using dots. Four independent replicates were performed. Values were represented using the mean ± SEM. (Scale bar, 5 μm.) Comparisons between groups were performed via the Mann-Whitney test. *P < 0.05, **P < 0.01, ***P < 0.001, and ****P < 0.0001 were considered statistically significant, whereas 'ns' indicates no significance.

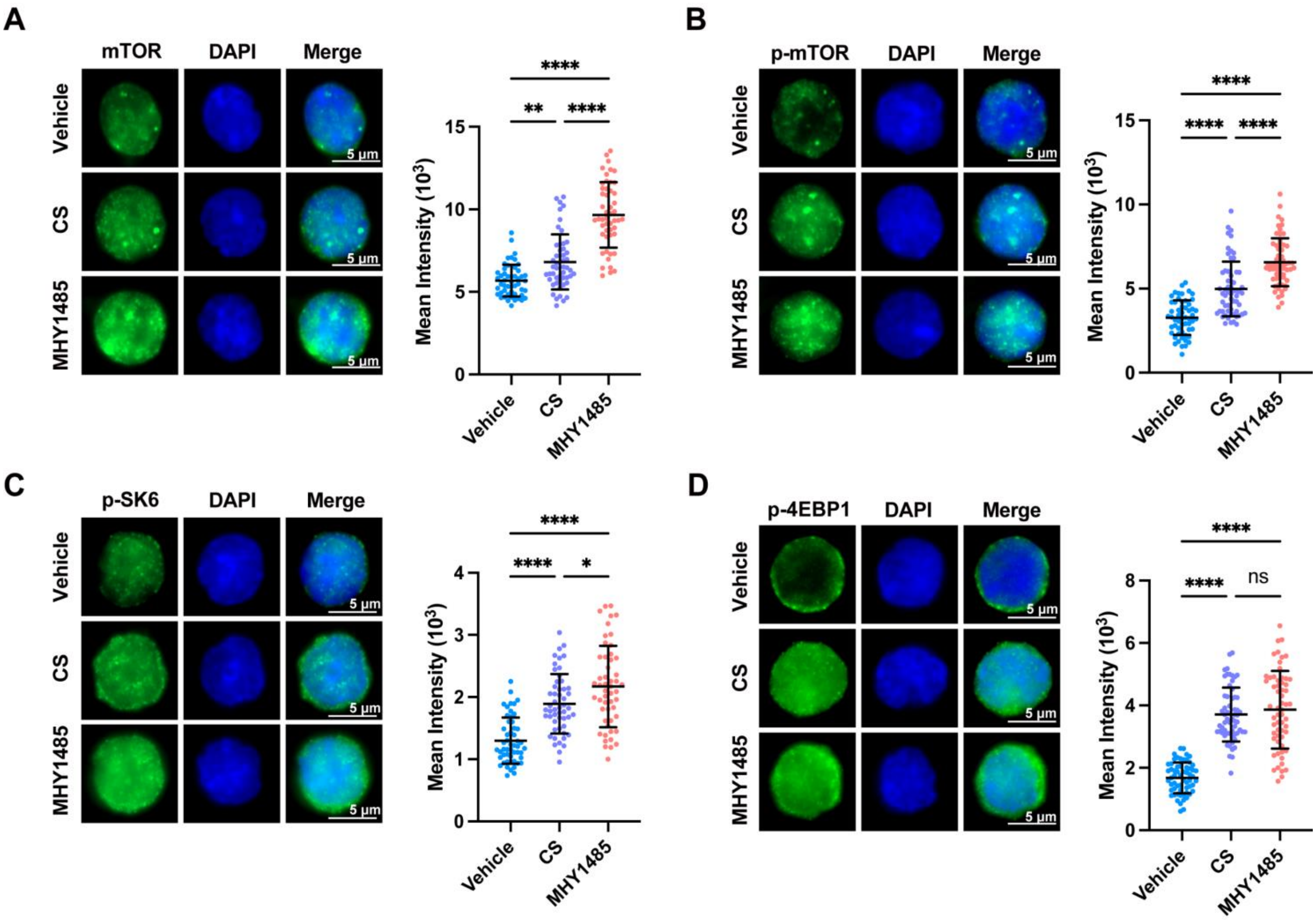

A
mTOR
DAPI
Merge
Vehicle
CS
MHY1485
5 µm
****
**
****
Mean Intensity (10³)
Vehicle
CS
MHY1485
B
p-mTOR
DAPI
Merge
****
****
****
C
p-SK6
DAPI
Merge
****
****
*
D
p-4EBP1
DAPI
Merge
****
****
ns

Figure S5. CS induces physiological restoration rather than hyperactivation of mTOR signaling in aged HSCs.

(**A-D**) Representative immunofluorescence images of total mTOR, phosphorylated mTOR (p-mTOR), p-S6K, and p-4EBP1 in HSCs sorted from aged mice treated with Vehicle, CS (25 mg/kg), or the mTOR agonist MHY1485 (5 mg/kg). Nuclei were counterstained with DAPI (blue). Quantification of the mean fluorescence intensity (MFI) for mTOR, p-mTOR, p-S6K, and p-4EBP1 (n = 4-5, each group). (Scale bar, 5 μm.) Data are presented as mean ± SEM. Each dot represents an individual cell (n≥30 cells per group). Statistical significance was determined using unpaired t-test. *P < 0.05, ***P < 0.001, ****P < 0.0001, ns: not significant.

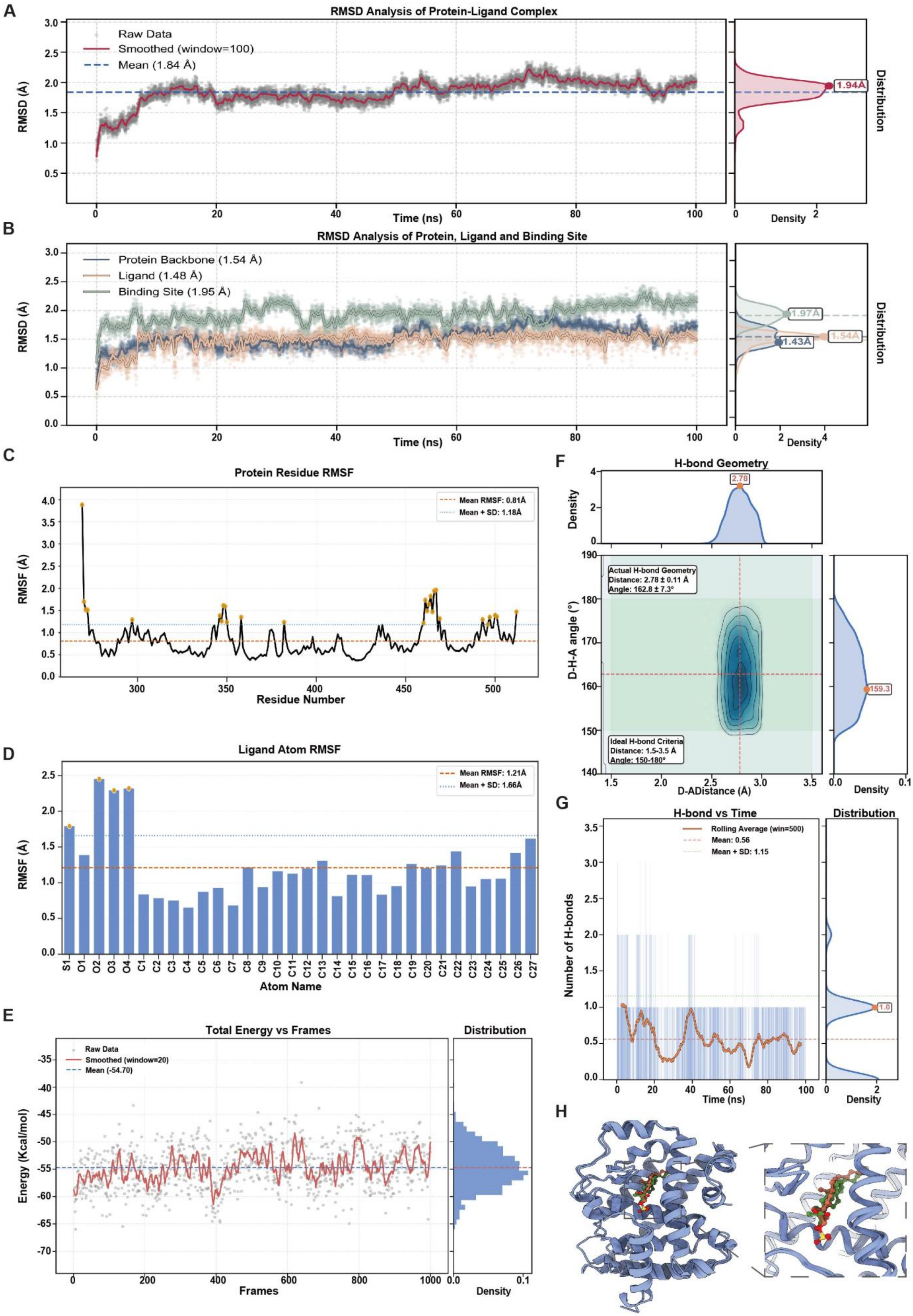

A
RMSD Analysis of Protein-Ligand Complex
Raw Data
Smoothed (window=100)
Mean (1.84 Å)
RMSD (Å)
Time (ns)
1.94Å
Density
Distribution
B
RMSD Analysis of Protein, Ligand and Binding Site
Protein Backbone (1.54 Å)
Ligand (1.48 Å)
Binding Site (1.95 Å)
RMSD (Å)
Time (ns)
1.97Å
1.54Å
1.43Å
Density
Distribution
C
Protein Residue RMSF
Mean RMSF: 0.81Å
Mean + SD: 1.18Å
RMSF (Å)
Residue Number
D
Ligand Atom RMSF
Mean RMSF: 1.21Å
Mean + SD: 1.66Å
RMSF (Å)
S1 O1 O2 O3 O4 C1 C2 C3 C4 C5 C6 C7 C8 C9 C10 C11 C12 C13 C14 C15 C16 C17 C18 C19 C20 C21 C22 C23 C24 C25 C26 C27
Atom Name
E
Total Energy vs Frames
Raw Data
Smoothed (window=20)
Mean (-54.70)
Energy (Kcal/mol)
Frames
Distribution
Density
F
H-bond Geometry
2.78
Density
Actual H-bond Geometry
Distance: 2.78 ± 0.11 Å
Angle: 162.8 ± 7.3°
Ideal H-bond Criteria
Distance: 1.5-3.5 Å
Angle: 150-180°
D-H-A angle (°)
D-ADistance (Å)
159.3
Density
G
H-bond vs Time
Rolling Average (win=500)
Mean: 0.56
Mean + SD: 1.15
Number of H-bonds
Time (ns)
Distribution
1.0
Density
H

Figure S6. Molecular dynamics (MD) simulation analysis of the RORA–cholesterol sulfate (CS) complex. (A) RMSD trajectory of the RORA–CS complex over the simulation period. The right panel displays the RMSD distribution histogram. **(B)** Comparative RMSD plots for the protein backbone (blue), ligand (orange), and binding site residues (green). The right panel shows the corresponding density plots. **(C)** RMSF analysis of RORA residues, where the x-axis represents residue indices and the y-axis indicates RMSF values (Å). The blue line denotes residue fluctuation, the red dashed line shows the average RMSF, and the blue dotted line marks the average plus one standard deviation. Residues with high flexibility are highlighted in yellow; different chains are color-coded. **(D)** RMSF values for individual atoms of the CS ligand. **(E)** Time-evolution of the total binding free energy, calculated by frame-wise estimation. The right panel shows the energy distribution. **(F)** Two-dimensional joint distribution of hydrogen bond geometry (donor–acceptor distance vs. angle). High-density regions (deep blue) indicate stable hydrogen bonding interactions. **(G)** Time-dependent frequency of hydrogen bond formation between CS and RORA. **(H)** Structural superimposition of the final MD-optimized conformations of RORA–CS and RORA–4ACD8 complexes. Detailed quantitative metrics are provided in Table S4.

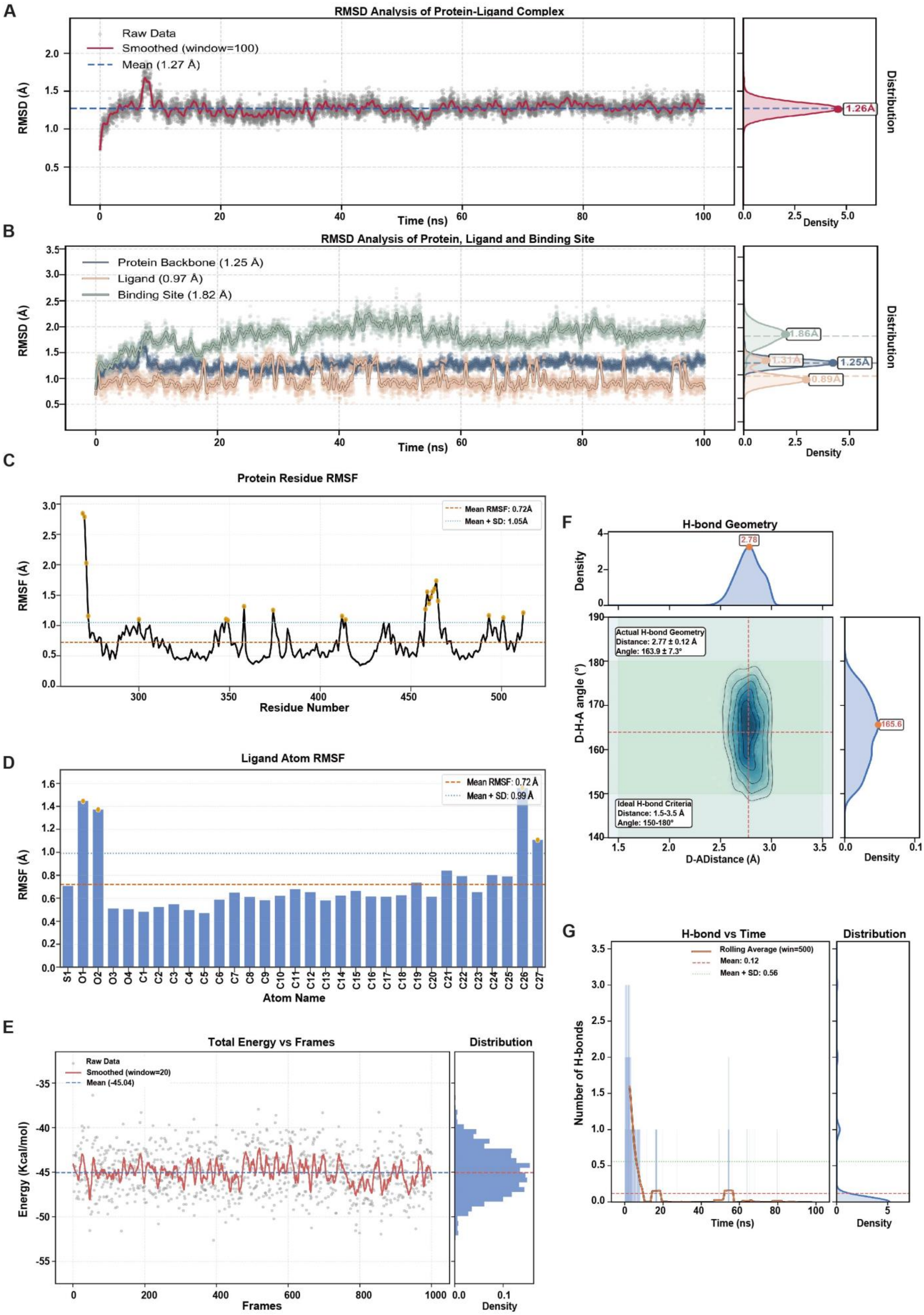

A
RMSD Analysis of Protein-Ligand Complex
Raw Data
Smoothed (window=100)
Mean (1.27 Å)
RMSD (Å)
Time (ns)
Distribution
1.26Å
Density
B
RMSD Analysis of Protein, Ligand and Binding Site
Protein Backbone (1.25 Å)
Ligand (0.97 Å)
Binding Site (1.82 Å)
1.86Å
1.31Å
1.25Å
0.89Å
C
Protein Residue RMSF
Mean RMSF: 0.72Å
Mean + SD: 1.05Å
RMSF (Å)
Residue Number
D
Ligand Atom RMSF
Mean RMSF: 0.72 Å
Mean + SD: 0.99 Å
Atom Name
E
Total Energy vs Frames
Raw Data
Smoothed (window=20)
Mean (-45.04)
Energy (Kcal/mol)
Frames
F
H-bond Geometry
2.78
Actual H-bond Geometry
Distance: 2.77 ± 0.12 Å
Angle: 163.9 ± 7.3°
Ideal H-bond Criteria
Distance: 1.5-3.5 Å
Angle: 150-180°
D-H-A angle (°)
D-ADistance (Å)
165.6
G
H-bond vs Time
Rolling Average (win=500)
Mean: 0.12
Mean + SD: 0.56
Number of H-bonds
Time (ns)

Figure S7. Molecular dynamics simulation analysis of the RORA–4ACD8 complex. (**A**) RMSD trajectory of the RORA–4ACD8 complex (positive control) showing structural stability over time. The right panel displays the RMSD distribution. (**B**) Component-wise RMSD analysis for the protein backbone, ligand, and binding site residues. (**C**) RMSF analysis of RORA residues bound to 4ACD8, with highly flexible regions highlighted in yellow. (**D**) RMSF values representing the atomic fluctuations of the 4ACD8 ligand. (**E**) Binding free energy trajectory of the RORA–4ACD8 complex. (**F**) Joint distribution map of hydrogen bond geometry. (**G**) Hydrogen bond count between RORA and 4ACD8 over the simulation course. Detailed quantitative simulation parameters and results are listed in Table S4.

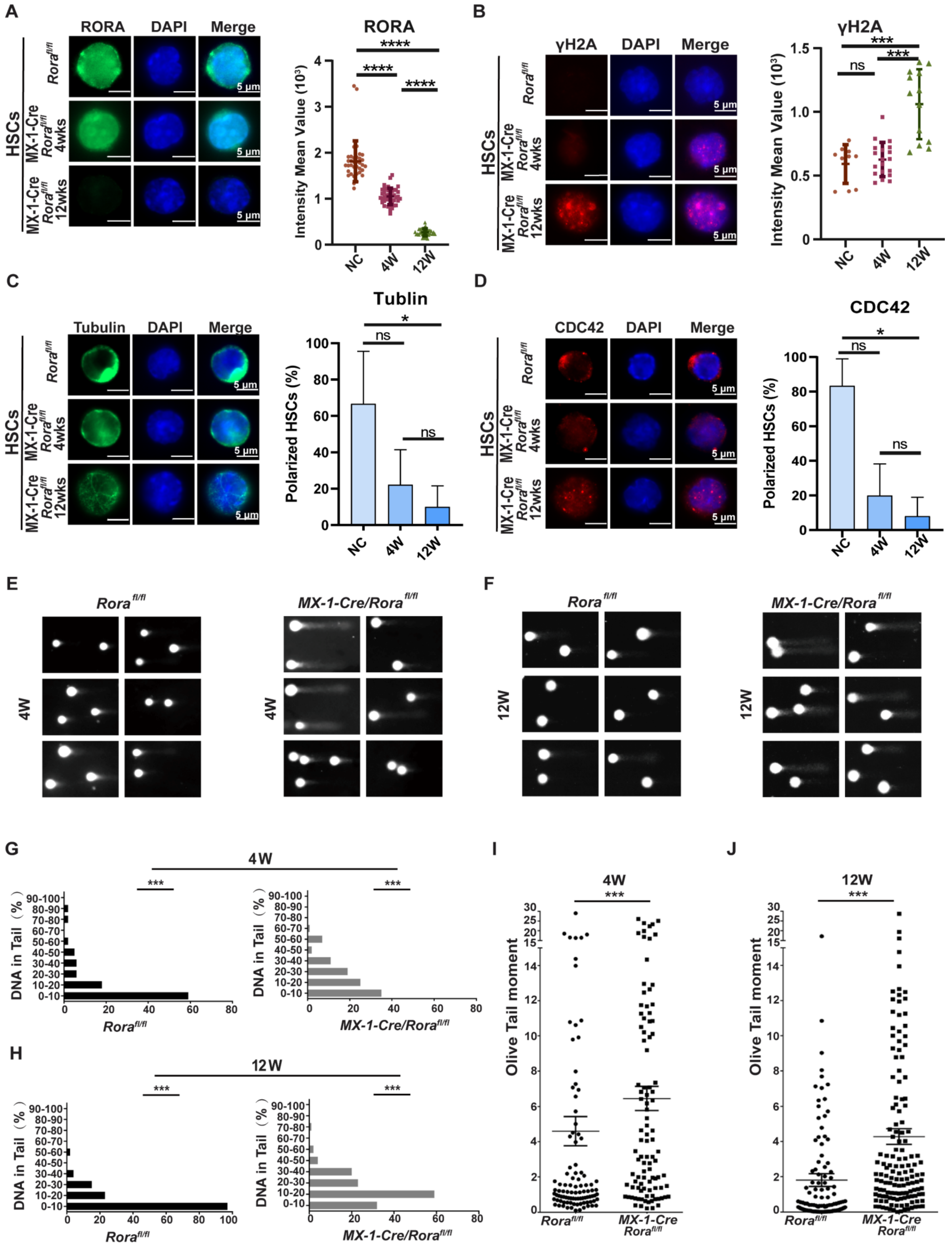
A
RORA
DAPI
Merge
HSCs
Rora fl/fl
MX-1-Cre Rora fl/fl 4wks
MX-1-Cre Rora fl/fl 12wks
5 μm
RORA
Intensity Mean Value (10³)
NC
4W
12W
****
B
γH2A
DAPI
Merge
γH2A
ns
***
C
Tubulin
DAPI
Merge
Tublin
Polarized HSCs (%)
*
ns
D
CDC42
DAPI
Merge
E
Rora fl/fl
MX-1-Cre/Rora fl/fl
4W
F
12W
G
4W
DNA in Tail (%)
90-100
80-90
70-80
60-70
50-60
40-50
30-40
20-30
10-20
0-10
H
12W
I
4W
Olive Tail moment
J
12W

Figure S8. Genetic deletion of *Rora* drives premature aging phenotypes. (A) Evaluation of RORA necessity via a conditional knockout model. Immunofluorescence validation showing significant and sustained depletion of RORA protein (green) in $Rora^{fl/fl}$*Mx-1-Cre* HSCs compared to controls at 4 and 12 weeks. (Scale bar, 5 µm.) **(B)** Quantification of DNA damage accumulation via γH2AX staining (red). The MFI of γH2AX foci progressively increased in *Rora*-deficient HSCs. (Scale bar, 5 µm.) **(C, D)** Analysis of HSC polarity. Representative images (left) and quantification (right) of polarized HSCs based on the asymmetric distribution of **(C)** Tubulin (green) and **(D)** CDC42 (red). (Scale bar, 5 µm.) **(E–J)** Comet assay assessing genomic instability. **(E, F)** Representative comet images of HSCs from control and *Rora*-deficient mice at **(E)** 4 weeks and **(F)** 12 weeks post-induction. **(G, H)** Quantification of the percentage of DNA in the tail at **(G)** 4 weeks and **(H)** 12 weeks. **(I, J)** Quantification of the Olive Tail Moment at **(I)** 4 weeks and **(J)** 12 weeks. Data are presented as mean ± SD. Each dot represents an individual cell. Statistical significance was determined using Mann–Whitney test. *P < 0.05, ***P < 0.001, ****P < 0.0001, ns: not significant.

Table S1. Detailed antibody panels and surface marker definitions

Cell type	Bio-markers
Lineage	Mac1, Gr1, Ter119, B220, Il7ra, Cd3, Cd4, Cd8
B	B220+
T	Cd3+
Myeloid	Gr-1+Mac-1+
Hematopoietic Stem and ProgenitorCell (HSPC)	LSKs. LinlowSca1+c-Kit+
Long-Term HSC (LT-HSC)	Linlowc-Kit+Sca-1+Cd48-Cd150+
Short-Term HSC (ST-HSC)	Linlowc-Kit+Sca-1+Cd48-Cd150-
Multi-Potent Progenitors (MPP)	Linlowc-Kit+Sca-1+Cd48+Cd150-
Common Myeloid Progenitors (CMP)	Linlowc-Kit+Sca-1- Cd16/32lowCd34high
Megakaryocyte-Erythroid Progenitor (MEP)	Linlowc-Kit+Sca-1- Cd16/32lowCd34low
Granulocyte-Monocyte Progenitor (GMP)	Linlowc-Kit+Sca-1- Cd16/32highCd34high

Table S2. Primary and Secondary Antibodies Targeting Key Proteins

Targets	Primary antibody	Secondary antibody
RORA	anti-RORA	anti-goat AMCA-conjugated antibody
Tubulin	anti-ALPHA Tubulin antibody	anti-rat AMCA-conjugated secondary antibody
CDC42	anti-CDC42 antibody	anti-rabbit DyLight5490-conjugated antibody
p-mTOR	anti-phospho-mTOR antibody	anti-rabbit DyLight5490-conjugated antibody
mTOR	anti-mTOR antibody	anti-rabbit DyLight5490-conjugated antibody
PARP1	anti- PARP1 antibody	anti-rabbit DyLight5490-conjugated antibody
ATM	anti-Atm antibody	anti-rat DyLight488-conjugated antibody
E2F1	anti- E2F1 antibody	anti-rabbit DyLight5490-conjugated antibody
